# A novel developmental critical period of orexinergic signaling in the primary visual thalamus

**DOI:** 10.1016/j.isci.2024.110352

**Published:** 2024-06-24

**Authors:** Anna M. Sanetra, Jagoda S. Jeczmien-Lazur, Kamil Pradel, Jasmin D. Klich, Katarzyna Palus-Chramiec, Marcelina E. Janik, Sylwia Bajkacz, Gabriela Izowit, Christian Nathan, Hugh D. Piggins, Alessio Delogu, Mino D.C. Belle, Marian H. Lewandowski, Lukasz Chrobok

**Affiliations:** 1 Department of Neurophysiology and Chronobiology, Institute of Zoology and Biomedical Research, Faculty of Biology, Jagiellonian University in Krakow, Krakow, Poland; 2 Institute for Systems Physiology, University of Cologne, Cologne, Germany; 3 Max-Delbrueck-Center for Molecular Medicine in the Helmholtz Association, Berlin, Germany; 4 Department of Glycoconjugate Biochemistry, Institute of Zoology and Biomedical Research, Faculty of Biology, Jagiellonian University in Krakow, Krakow, Poland; 5 Department of Inorganic Chemistry, Analytical Chemistry and Electrochemistry, Faculty of Chemistry, Silesian University of Technology, Gliwice, Poland; 6 The Biotechnology Centre, Silesian University of Technology, Gliwice, Poland; 7 Division of Neuroscience, School of Biological Sciences, Faculty of Biology, Medicine and Health, The University of Manchester, Manchester, UK; 8 University of Exeter Medical School, Hatherly Labs, Streatham Campus, Prince of Wales Road, Exeter, Devon, UK; 9 School of Physiology, Pharmacology, and Neuroscience, Faculty of Health and Life Sciences, University of Bristol, Bristol, UK; 10 Department of Basic and Clinical Neuroscience, Institute of Psychiatry, Psychology and Neuroscience, King’s College London, London, UK

**Keywords:** Optical signal processing, Neuroscience, Behavioral neuroscience

## Abstract

The orexinergic system of the lateral hypothalamus plays crucial roles in arousal, feeding behavior, and reward modulation. Most research has focused on adult rodents, overlooking orexins’ potential role in the nervous system development. This study, using electrophysiological and molecular tools, highlights importance of orexinergic signaling in the postnatal development of the rodent dorsolateral geniculate nucleus (DLG), a primary visual thalamic center. Orexin activation of DLG thalamocortical neurons occurs in a brief seven-day window around eye-opening, concurrent to transient OX_2_ receptor expression. Blocking OX_2_ receptors during this period reduces sensitivity of DLG neurons to green and blue light and lowers spontaneous firing rates in adulthood. This research reveals critical and temporally confined role of orexin signaling in postnatal brain development, emphasizing its contribution to experience-dependent refinement in the DLG and its long-term impact on visual function.

## Highlights

• Sensitivity to OXA was studied in the rodent dorsolateral geniculate (DLG) nucleus
• DLG neurons respond to OXA within a week-long period around eye-opening
• This coincides with developmental window in OX_2_ receptor expression
• Blocking OX_2_ receptors in pups impairs visual processing in adulthood

## Graphical abstract

**Figure:**
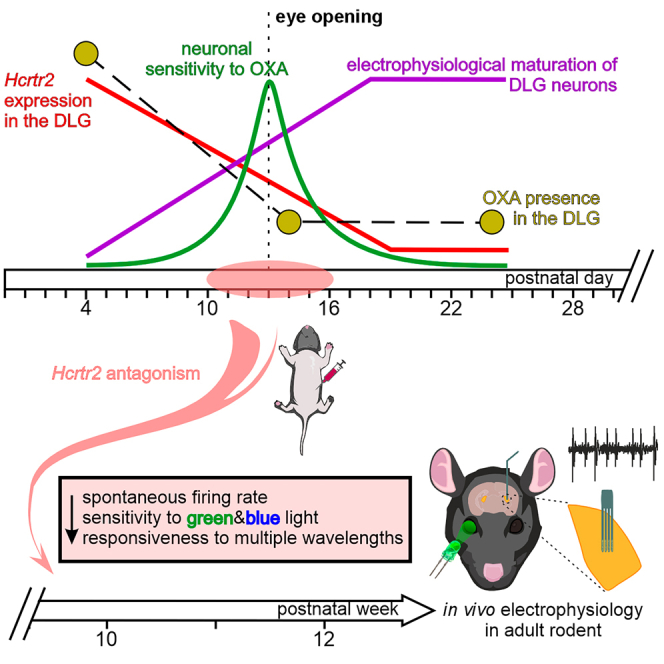

## Introduction

The development of the mammalian brain is sustained over the postnatal period. This is especially crucial for sensory systems which in the complete or partial absence of key environmental stimuli in particular periods of ontogeny (called critical periods) are dysfunctional in adulthood.^1^^,^^2^ An exemplar of this is the primary visual system, a complex and highly specialized set of neuronal structures which detect, process, and relay light information to form a reliable representation of the external world. The simplest primary visual pathway includes the retina, dorsolateral geniculate nucleus (DLG) of the thalamus, and primary visual cortex (V1). With its dependence on environmental stimuli (light), the visual system shares some characteristics with other sensory systems, undergoing dramatic postnatal changes, including synaptic pruning and potentiation, neuronal elimination, neurophysiological maturation, and overall shift in gene expression profiles.^2^^,^^3^ Eye opening, which in mice and rats occurs around postnatal day (PD)12–14 is pivotal in this developmental program.^2^^,^^4^

Previous studies from our laboratory and of others have shown that the orexinergic neurons of the lateral hypothalamus which compose a major brain arousal circuit,^5^^,^^6^^,^^7^ are important modulators of neuronal activity in the extended visual system. The two forms of orexin, orexin A (OXA) and orexin B (OXB), act via their cognate receptors (orexin receptor 1 and 2: OX_1_ and OX_2_ receptor, respectively) to alter the activity in non-image forming visual centers, including the intergeniculate leaflet (IGL), ventrolateral geniculate nucleus (VLG), olivary pretectal nucleus (OPN), superior colliculus (SC), and suprachiasmatic nucleus of the hypothalamus (SCN).^8^^,^^9^^,^^10^^,^^11^^,^^12^^,^^13^^,^^14^ However, the sensitivity of the image-forming DLG neurons to orexin has been unclear.

The DLG is only sparsely innervated by the orexinergic fibers.^15^^,^^16^^,^^17^ Initial studies on adult rats and mice did not detect significant orexin receptor expression in this structure.^18^^,^^19^^,^^20^ Consistent with this, patch-clamp recording studies in the thalamus of two to three week old rats *ex vivo* reported no sensitivity to orexins in the DLG.^21^^,^^22^ In contrast, our earlier patch-clamp experiments *ex vivo* on approximately two-week-old rats showed that ∼75% of recorded DLG thalamocortical neurons were robustly excited by orexins, acting via the OX_2_ receptors.^15^ Interestingly, however, these notable effects of OXA on neuronal activity in the DLG were not replicated in adult rats, either *in vivo*^16^ or *ex vivo*.^8^

The orexin system is functional during brain development, including embryonic and early postnatal periods. The expression of prepro-orexin in mice is detected at embryonic day (ED)12, and orexin-immunoreactive fibers are detected throughout the prepartum brain.^23^^,^^24^ Similarly, developmental changes in the expression of orexin receptors are described in several brain areas, including the hypothalamus, hypoglossal nucleus, and cerebellum.^23^^,^^25^^,^^26^ However, the role of orexin signaling in the functional ontogeny of brain centers, including the visual system, remains unknown.

Here, we aimed to address this important knowledge-gap by investigating the role of orexin signaling in the development of the rodent thalamocortical DLG neurons, and the consequences of impairing this peptidergic signal for visual processing in adult rats. Using patch-clamp and multi-electrode array (MEA) electrophysiology *ex vivo*, we first show that there is a short 7-day developmental window in DLG neuronal responsiveness to orexin which peaks at the day of eye opening. Additionally, we revealed that this transient period of sensitivity to OXA concurs with neurophysiological maturation of studied DLG neurons. Next, we combined electrophysiology with molecular tools, *in situ* hybridization, and chromatography, to show that the enhanced responsiveness to orexin occurs in parallel with the developmental expression of OX_2_ receptor in the DLG. Finally, through *in vivo* electrophysiological recordings, we present evidence that the blockade of OX_2_ receptors during this developmental period results in profound disruptions to light-evoked responsivity in the adult DLG. Our studies are performed on both mice and rats to provide evidence that this developmental program is conserved between rodent species. Altogether, our study is the first to demonstrate the pivotal and temporally confined role of orexin signaling in postnatal brain development.

## Results

### OXA evokes firing of DLG neurons *ex vivo* in a concise developmental window

**Figure 1:**
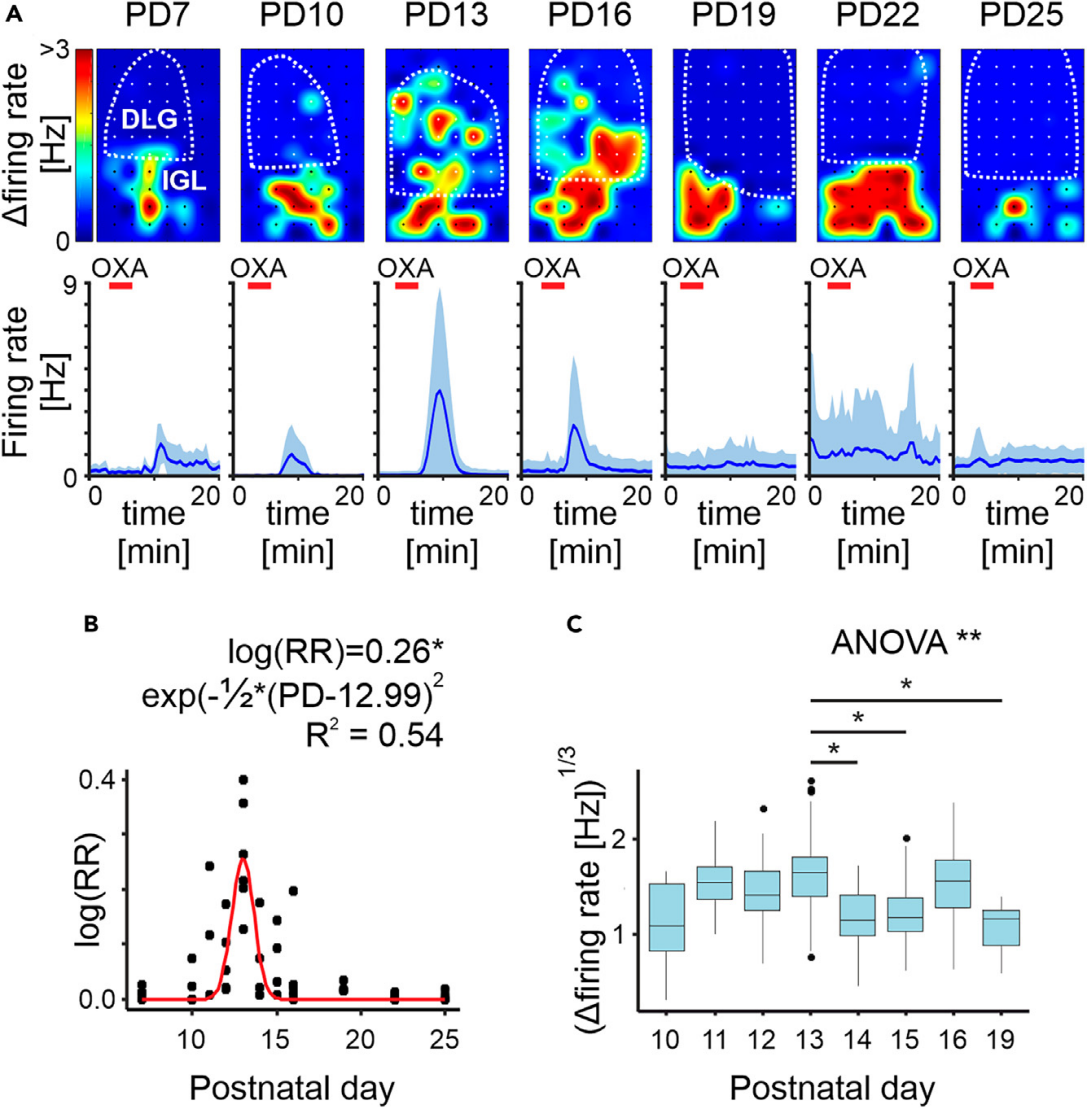
OXA evokes firing of rat DLG neurons *ex vivo* in a concise developmental window (A) Spatial heatmaps of a representative brain slice for each age group with a color-coded change in firing rate (multi-unit activity at each recording electrode) during OXA application (200 nM, *top row*) together with average activity plots showing temporal changes in the single-unit neuronal activity during all experiments (*bottom row*). Red bar depicts the time of OXA treatment. Note the persistence of the effect in the IGL for all age groups, and clear age-dependence in the DLG. (B) Changes in the responsiveness to OXA across all studied postnatal days (PDs) presented as a ratio of responsive neurons over the number of electrodes within the DLG (RR – responsiveness ratio) with each dot representing a brain slice. Fitted Gaussian equation in a form of $\kappa \times \exp\;(-\frac{{\left(\text{PD}-\mu \right)}^{2}}{2\sigma^{2}})$ pointed to PD13 as the developmental time-window where most of the responses were observed (μ = 12.99 ± 0.13, *t*_46_ = 101.24, *p* < 0.0001; к = 0.26 ± 0.029, *t*_46_ = 8.8, *p* < 0.0001; σ = 0.65 ± 0.09, *t*_46_ = 7.39, *p* < 0.0001). (C) Changes in the response amplitude to OXA analyzed between PD10-19 (due to a very low number of responses at PD7, 22 and 25). The linear mixed effects model contained PD as a fixed factor (*F*_7,22_ = 4.47, *p* = 0.0032) and rat as a random intercept (SD = 0.14), nesting observations from the same animal. Largest response was observed at PD13 (PD13-PD14: *t*_22_ = 3.45, *p* = 0.04; PD13-PD15: *t*_22_ = 3.61, *p* = 0.028; PD13-PD19: *t*_22_ = 3.8, *p* = 0.018). ∗*p* < 0.05, ∗∗*p* < 0.01.

Over the last two decades, the role of orexins in the thalamus was hypothesized to be functionally segregated; orexins were found to selectively modulate neuronal activity of ‘non-specific’ thalamic nuclei, but not those containing thalamocortical neurons involved in processing ‘specific’ sensory information.^21^ In accordance with this, previous reports from our group showed that OXA is not a potent modulator of neuronal activity within the adult rat DLG, with this peptide activating less than 10% of recorded units both *in vivo* as well as *ex vivo*.^15^^,^^27^ However, results of patch-clamp *ex vivo* studies performed on younger animals are inconsistent.^15^^,^^21^^,^^22^ Thus, with the use of MEA recording technology, we initially evaluated neuronal responsiveness to OXA in 49 thalamic brain slices containing the LGN prepared from 43 rat pups culled between PD7 and PD25 (3–5 animals per group). Brain slices were positioned on the MEAs so that recordings were made from majority of the DLG as well as the subjacent IGL where, in pups and adults, OXA is reported to alter spontaneous and evoked activity.^8^^,^^12^ Strikingly, the application of OXA (200 nM) potently evoked changes in neuronal firing in the DLG within a concise time window between PD10 and PD16 (Figures 1A and 1B). On the day of eye-opening (PD13), a clear peak in both the density of responsive units (μ = 12.99 ± 0.13, *t*_46_ = 101.24, *p* < 0.0001, *n* = 49; Figure 1B) and the response amplitude (*F*_7,22_ = 4.47, *p* = 0.0032, *n* = 450; Figure 1C) was observed. By contrast, IGL neurons did not alter in sensitivity and were responsive to OXA throughout all developmental stages tested (Figure 1A).

**Figure 2:**
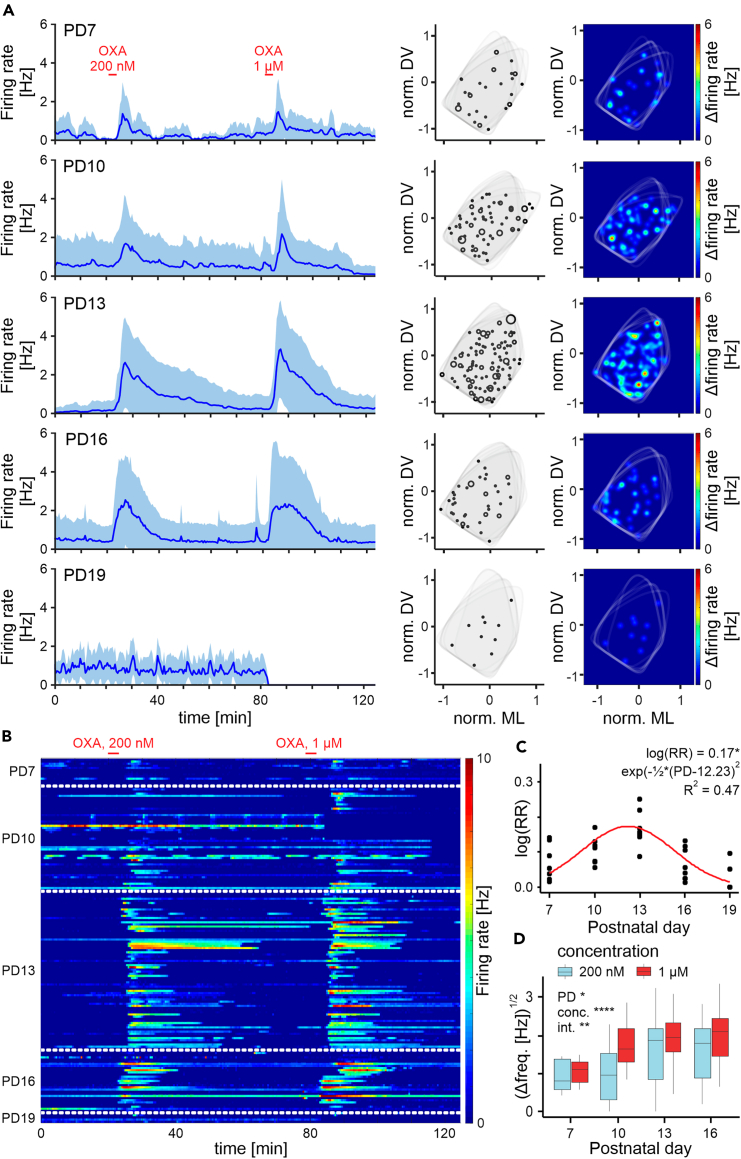
OXA evokes firing of mouse DLG neurons *ex vivo* in a concise developmental window (A) Average single-unit activity plots showing temporal changes in the neuronal activity during all experiments (*left*), as well as their spatial distribution (*right*). (B) Color-coded temporal heatmap presenting both the intensity of the response as well as its prevalence for each age group. (C) Changes in the responsiveness to OXA across all studied PDs presented as a ratio of responsive neurons over the number of electrodes within the DLG (RR – responsiveness ratio) for each brain slice (each slice represented by a dot). Fitted a Gaussian equation in a form of $\kappa \times \exp\;\left(-\frac{{\left(\text{PD}-\mu \right)}^{2}}{2\sigma^{2}}\right)$ pointed to PD12 as the time of the most frequently observed response (μ = 12.23 ± 0.38, *t*_34_ = 31.8, *p* < 0.0001; к = 0.17 ± 0.019, *t*_34_ = 9.26, *p* < 0.0001; σ = 3.06 ± 0.39, *t*_34_ = 7.87, *p* < 0.0001). (D) Changes in the amplitude of the response to OXA analyzed between PD7-16 (due to a very low number of responses at PD19). The linear mixed effects model contained PD (*F*_3,11_ = 4.95, *p* = 0.021) and OXA concentration (*F*_1,125_ = 63.63, *p* < 0.0001) as fixed factors, as well as an interaction between them (*F*_3,125_ = 4.81, *p* = 0.0033). Random effects included the animal as a random intercept (SD = 0.00063), another random intercept for each cell (SD = 2.27), and a random slope for each neuron, pairing the data between the different concentrations (SD = 1.64). *Post hoc* tests revealed an increase in the response amplitude to the lower (200nM) OXA concentration between PD10 and PD13 (*t*_11_ = −3.48, *p* = 0.023), and a significantly lower response at PD7 to the higher (1μM) OXA concentration (PD7-PD13: *t*_11_ = −3.42, *p* = 0.025; PD7-PD16: *t*_11_ = 3.57, *p* = 0.02). Differences in the sensitivity to OXA concentration were present in all groups except PD7 (*t*_125_ = −0.71, *p* = 0.48; PD10: *t*_125_ = −6.83, *p* < 0.0001; PD13: *t*_125_ = −4.1, *p* = 0.0001; PD16: *t*_125_ = −3.74, *p* = 0.0003). ∗*p* < 0.05, ∗∗*p* < 0.01, ∗∗∗∗*p* < 0.0001. See also Figure S1.

To test the possibility that the developmental period of OXA action in the DLG occurs in other rodent species, we prepared 37 thalamic slices from 19 mice (3–4 animals per group) and with MEA recordings assessed how OXA influenced DLG activity between PD7 and PD19. Few responses to OXA were detected at PD7, however, robust responsiveness was found between PD10 and PD16 (Figures 2A–2C), with peak responsiveness at PD12 (extrapolation with bell-curve fitting; μ = 12.23 ± 0.38, *t*_34_ = 31.8, *n* = 37, *p* < 0.0001; Figure 2C); the average day of eye-opening for mice. The actions of OXA appeared dose dependent as the amplitude of response increased from a 200 nM treatment to 1 μM (*F*_1,125_ = 63.63, *p* < 0.0001, *n* = 129; Figure 2D). This suggests a receptor-specific action of this peptide. Interestingly, this dose-related difference in response was most substantial at PD10 (*t*_125_ = 6.83, *p* < 0.0001). This was not observed in mouse IGL neurons, which similar to recordings in rat IGL, were responsive to OXA treatment in all slices across age groups. The large recording area provided by a 120-channel recording array additionally covered the neighboring medial geniculate nucleus (MGN) – a thalamic center of the auditory system. Interestingly, no neuronal responses to either 200 nM or 1 μM OXA were noted in the MGN at any of the developmental timepoints tested (Figure S1). Together, these results provide evidence that OXA dose-relatedly affects neuronal activity in the rodent DLG in a concise developmental window of approximately one week in duration and centered around the day of eye-opening. Further, this dataset points to a potential specificity of developmental action or orexinergic system on the visual, over the other sensory systems.

### OXA modulates the optic nerve input to the DLG

**Figure 3:**
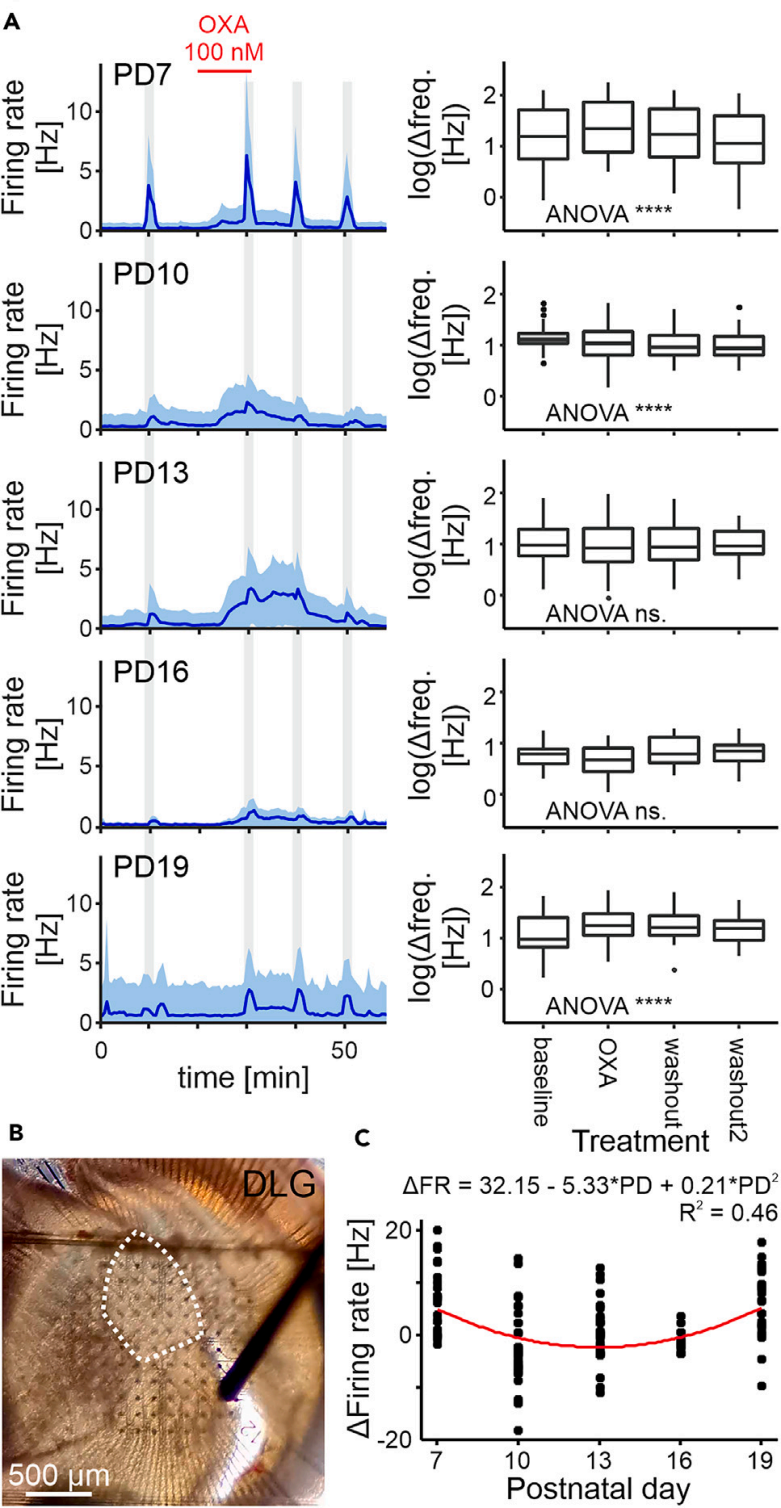
OXA modulates the optic input to the DLG (A) Average single-unit activity plots for each age group studied (*left panels*) with an analysis of the change in firing rate before and during tonic OXA application, as well as during a washout (*right panels*). Gray shadings indicate the times of the optic tract stimulation. For each age group, we fitted a linear model with postnatal day (PD) as a fixed factor and random factors in a form of a random intercept for each animal, and a random intercept for each cell with a random slope for the successive stimulations. OXA was shown to influence neuronal responsiveness to the optic tract stimulation at PD7, 10 and 19 (PD7: *F*_3,99_ = 17.68, *p* < 0.0001, *n* = 34; PD10: *F*_3,93_ = 7.41, *p* = 0.0002, *n* = 32; PD13: *F*_3,87_ = 0.36, *p* = 0.78, *n* = 30; PD16: *F*_3,36_ = 2.54, *p* = 0.07, *n* = 13; PD19: *F*_3,69_ = 6.75, *p* = 0.0005, *n* = 24). ∗∗∗∗*p* < 0.0001. (B) Representative photograph of the recording electrode matrix in the thalamus and the stimulating electrode on the optic nerve. The area of the DLG is delineated. (C) For the analysis of the difference in the response to the optic tract stimulation before and during tonic OXA application we fitted a quadratic function in a form of a + b × PD + c × PD^2^ (a = 32.15 ± 10.2, *t*_109_ = 3.15, *p* = 0.0021, b = −5.33 ± 1.73, *t*_14_ = −3.09, *p* = 0.0081, c = 0.21 ± 0.067, *t*_14_ = 3.06, *p* = 0.0085, *n* = 126), with a minimum value of −2.35 at PD = 12.95.

To build on these observations, we next investigated whether OXA influences DLG neuronal activity selectively evoked by stimulating optic nerve input to this structure. Thalamic slices were made from 17 mice across PD7 to PD19 (3–4 animals per group, *n* = 2 at PD19). Taking advantage of our 2-well MEA system, these experiments were performed simultaneously to those described above, using another set of slices from the same animals. In this set of recordings, OXA (100 nM) was continuously applied for 12 min while trains of electrical pulses (*n* = 50, frequency: 0.5 Hz, duration: 200 μs, amplitude: 500 μA) were delivered prior, during, a following the application (Figures 3A and 3B). Interestingly, the application of OXA significantly changed the response to optic tract stimulation at PD7 (*F*_3,99_ = 17.68, *p* < 0.0001, *n* = 34), PD10 (*F*_3,93_ = 7.41, *p* = 0.0002, *n* = 32), and PD19 (*F*_3,69_ = 6.75, *p* = 0.0005, *n* = 24), but not at the developmental time points at which the large magnitude responses to the peptide occurred [PD13 (*F*_3,87_ = 0.36, *p* = 0.78, *n* = 30) or PD16 (*F*_3,36_ = 2.54, *p* = 0.0.07, *n* = 13; Figure 3A)]. Thus, the orexinergic modulation of responses to the optic tract stimulation across the age groups can be fitted into a U-shaped curve (PD^2^: *F*_1,14_ = 9.35, *p* = 0.0085; Figure 3C). This dataset suggests that OXA differentially modulates the optic nerve input to the DLG during defined time points in the development of the visual system.

### Neurophysiological properties of DLG neurons mature postnatally at the time of their responsiveness to OXA

**Figure 4:**
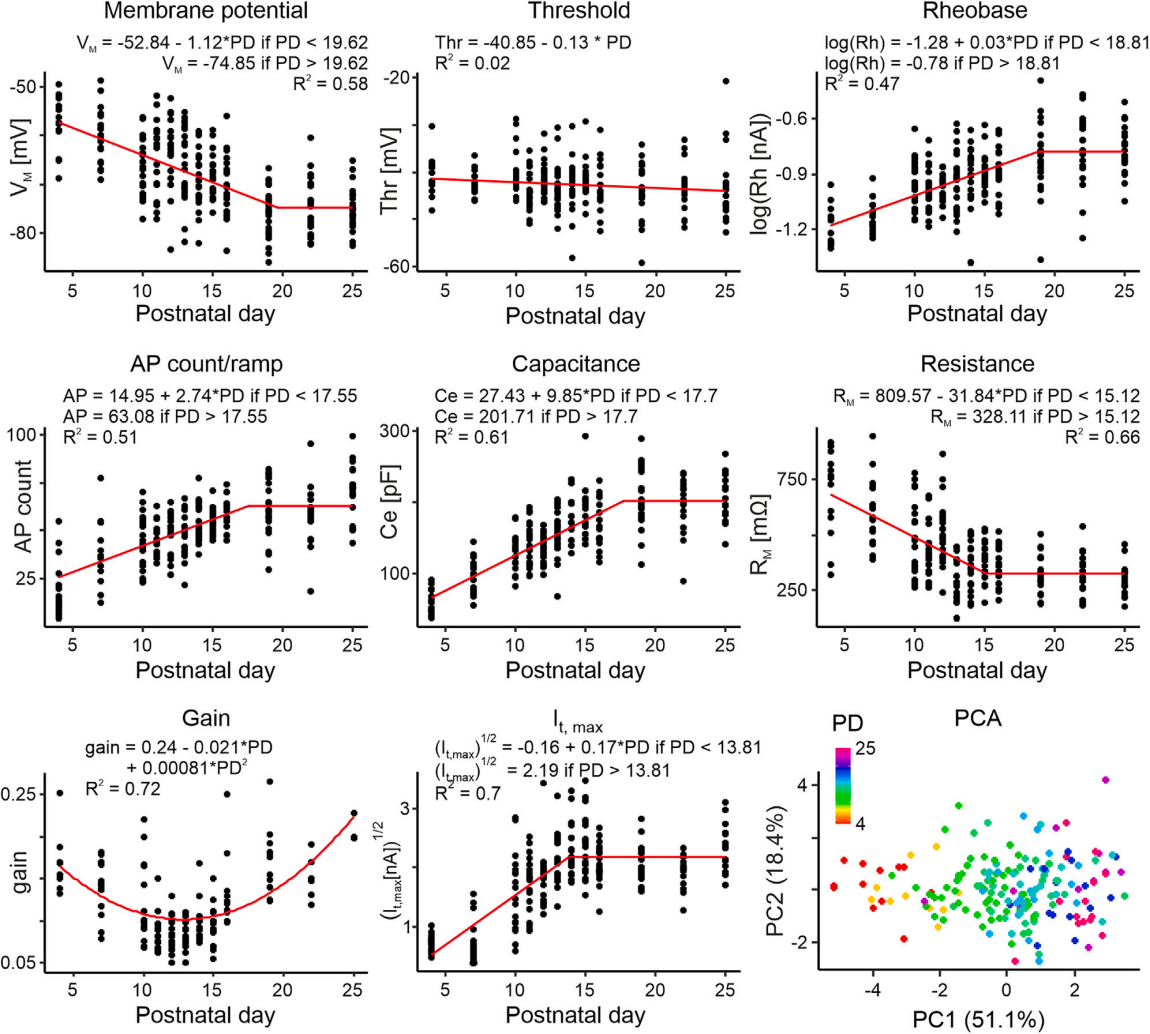
Neurophysiological properties of DLG neurons mature postnatally Mixed effects models were fitted for each electrophysiological parameter analyzed, with observations nested in an animal included as a random intercept. Majority of these parameters followed a linear-plateau fit in a form of a + b ∗x when x < jp, and a + b ∗ jp when x > jp (jp - junction point): membrane potential (a = −52.84 ± 1.85, *t*_192_ = −28.6, *p* < 0.0001, b = −1.12 ± 0.14, *t*_49_ = −7.88, *p* < 0.0001, jp = 19.62 ± 1.53, *t*_49_ = 12.81, *p* < 0.0001, SD_rat_ = 2.66, *n* = 244), rheobase (a = −1.28 ± 0.04, *t*_192_ = −31.85, *p* < 0.0001, b = 0.027 ± 0.0033, *t*_49_ = 8.03, *p* < 0.0001, jp = 18.81 ± 1.24, *t*_49_ = 15.2, *p* < 0.0001, SD_rat_ = 0.034, *n* = 244), action potential (AP) count (a = 14.95 ± 4, *t*_166_ = −3.73, *p* = 0.0003, b = 2.74 ± 0.33, *t*_49_ = 8.38, *p* < 0.0001, jp = 17.55 ± 1.07, *t*_49_ = 16.39, *p* < 0.0001, SD_rat_ = 3.64, *n* = 218), capacitance (a = 27.43 ± 9.08, *t*_172_ = 3.02, *p* = 0.0029, b = 9.85 ± 0.75, *t*_48_ = 13.12, *p* < 0.0001, jp = 17.7 ± 0.71, *t*_48_ = 24.85, *p* < 0.0001, SD_rat_ = 4.62, *n* = 223), resistance (a = 809.57 ± 50.62, *t*_170_ = 15.99, *p* < 0.0001, b = −31.84 ± 4.42, *t*_48_ = −7.21, *p* < 0.0001, jp = 15.12 ± 1.01, *t*_48_ = 14.98, *p* < 0.0001, SD_rat_ = 70.78, *n* = 221) and maximum amplitude of T-type calcium current (I_t_) (a = −0.16 ± 0.21, *t*_155_ = −0.77, *p* = 0.44, b = 0.17 ± 0.02, *t*_48_ = 8.3, *p* < 0.0001, jp = 13.81 ± 0.72, *t*_48_ = 19.3, *p* < 0.0001, SD_rat_ = 0.23, *n* = 206). Threshold appeared linear (a = −40.85 ± 0.86, *t*_192_ = −47.23, *p* < 0.0001, b = −0.13 ± 0.056, *t*_50_ = −2.22, *p* = 0.031, SD_rat_ = 0.00043, *n* = 244). However, the fit of the model was very poor, as indicated by low R^2^ value (R^2^ = 0.02). The only parameter not linearly changing at any point was the gain, for which we fitted a quadratic function in a form of a + b × PD + c × PD^2^, with a minimum value of 0.1 at PD = 12.82 (a = 0.24 ± 0.024, *t*_106_ = 9.88, *p* < 0.0001, b = −0.021 ± 0.0036, *t*_43_ = −5.76, *p* < 0.0001, c = 0.00081 ± 0.00013, *t*_43_ = 6.25, *p* < 0.0001, SD_rat_ = 0.024, *n* = 152). The last graph presents the results from the principal component analysis (PCA), which included all parameters except the gain. See also Figure S2 and Figure S3.

From the retina to visual cortex, the rodent visual system undergoes notable changes at distinct periods through postnatal development to attain the mature state around the fifth postnatal week.^2^ To determine how the fundamental neurophysiological properties of rat DLG neurons change over development and, in particular, around the time of eye-opening, we made patch-clamp recordings from DLG neurons in thalamic slices prepared from 52 rats (neurons were assessed on PD4, PD7, PD10, PD11, PD12, PD13, PD14, PD15, PD16, PD19, PD22, and PD25; 3–5 animals per group). Current-clamp recordings (holding current = 0) revealed that the membrane potential of DLG neurons hyperpolarized from −57.73 [confidence intervals: −61.17, −54.29] mV at PD4 to reach a mature-like state around PD20 (PD19: −75.67 [-77.88, −73.47] mV; jp = 19.62 ± 1.53, *t*_49_ = 12.81, *p* < 0.0001, *n* = 244; Figure 4). To evaluate if threshold for triggering action potentials varied over development, a current ramp (duration: 1s, amplitude: 1 nM) was applied from a set membrane potential (−65 mV; Figure S2A). Over PD4-25, the action potential threshold did not appear to alter (PD4: −40.87 [-43.10, −38.64] mV, PD25: −43.73 [-47.02, −40.43] mV). Indeed, though the slope of a regression function fitted on the threshold over development was significant (*t*_50_ = 2.22, *p* = 0.031, *n* = 244), this fitted model did not explain the variation (R^2^ = 0.02). By contrast, the rheobase increased with age, from 0.064 [0.056, 0.073] nA at PD4, to stabilize at 0.15 [0.13, 0.19] nA around PD19 (jp = 18.81 ± 1.24, *t*_49_ = 15.2, *p* < 0.0001, *n* = 244; Figure 4). The number of action potentials elicited on the ramp also increased with age, reaching a plateau around PD18 (from 21.07 [12.34, 29.81] at PD4 to 62.09 [55.51, 68.67] at PD19; jp = 17.55 ± 1.07, *t*_49_ = 16.39, *p* < 0.0001, *n* = 218; Figure 4). Next, to measure capacitance and membrane resistance, a current step protocol (from −150 pA to +150 pA, increment: 10 pA, duration: 0.5 s) was performed, also induced from the same set membrane potential for each cell tested (−65mV; Figure S2B). Responses to hyperpolarizing steps demonstrated that the capacitance of tested cells gradually increased from 60.78 [50.81, 70.74] pF at PD4, reaching a steady state around PD18 (PD19: 204.79 [184.38, 225.19] pF; jp = 17.7 ± 0.71, *t*_48_ = 24.85, *p* < 0.0001, *n* = 223), whereas the membrane resistance decreased until PD15 (from 681.50 [571.99, 791.01] MΩ at PD4 to 379.03 [341.23, 416.83] mΩ at PD15; jp = 15.12 ± 1.01, *t*_48_ = 14.98, *p* < 0.0001, *n* = 221; Figure 4). Intriguingly, a gain in action potential generation on consecutive steps (a measure of neuronal excitability) did not follow such a linear-plateau profile and instead fitted a parabolic curve function with a minimal value at PD13 (0.082 [0.073, 0.091]; Figure 4). The T-type calcium current is critical for the burst firing of DLG neurons, and we used a voltage-clamp protocol to measure this important parameter^28^^,^^29^ [neurons were voltage clamped at −75mV and a voltage step protocol (from −95mV to −38mV, increment: 3 mV, duration: 200 ms) applied]. The linear-plateau model was fitted and indicated that the amplitude of T-type current increased with age, from 0.53 [0.41, 0.67] nA at PD4, reaching a plateau around PD14 (2.29 [2.06, 2.52] nA; jp = 13.81 ± 0.72, *t*_48_ = 19.3, *p* < 0.0001, *n* = 206; Figure 4). Altogether, these results show that DLG neurons exhibit profound neurophysiological changes during the postnatal developmental window of their sensitivity to OXA.

These recordings indicate a developmental progression in the fundamental properties of DLG neurons and a subsequent principal component analysis (PCA) of all parameters described above (except for gain, which did not change linearly at any time point), revealed clear clustering of neurons within their developmental groups (Figure 4). Indeed, the first principal component described 51.1% of the variance indicating neurophysiological parameters of the DLG mature and stabilize around PD18 (jp = 17.59 ± 0.86, *t*_47_ = 20.36, *p* < 0.0001, *n* = 182; Figure S3).

**Figure 5:**
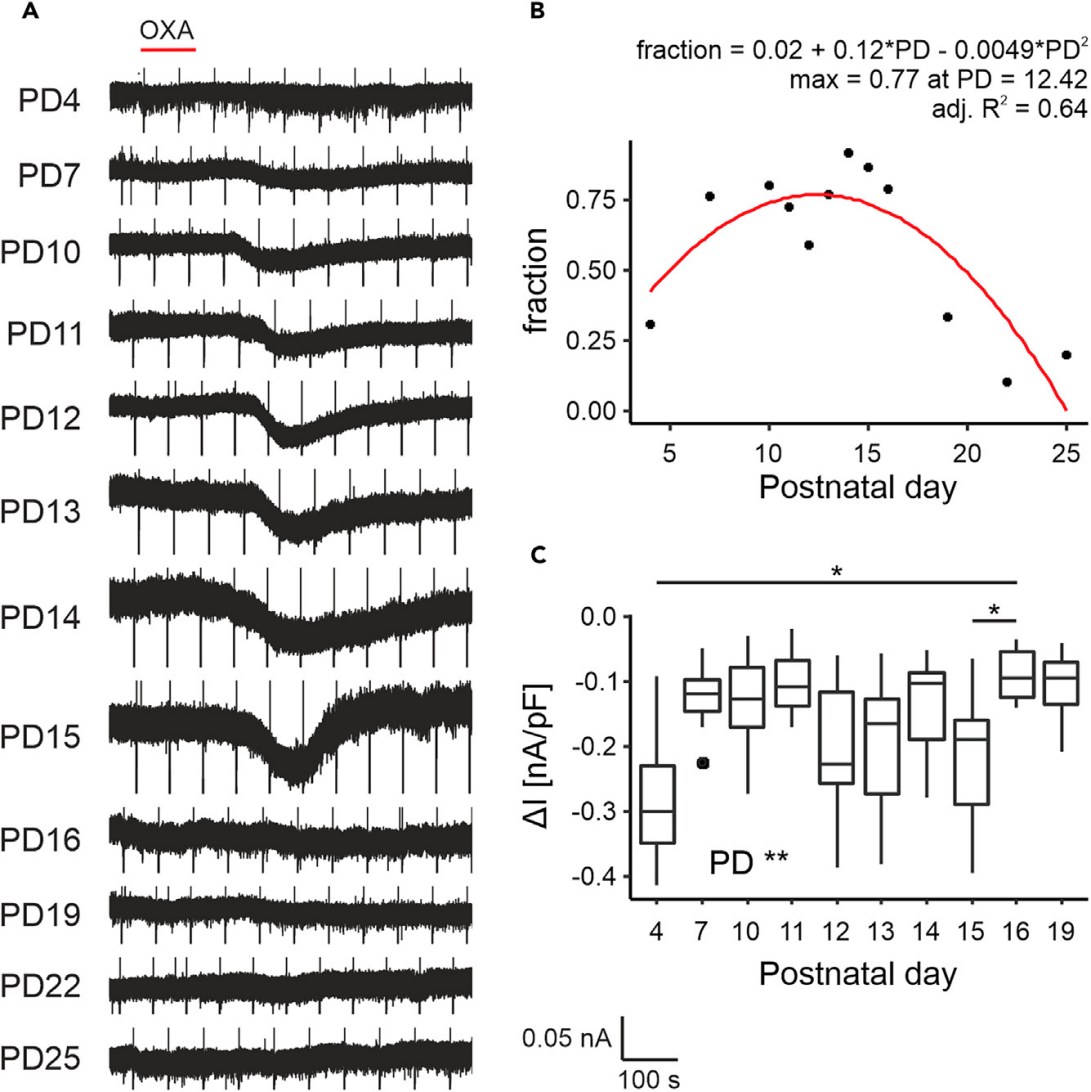
OXA evokes inward current in the rat DLG neurons *ex vivo* in a concise developmental window (A) Representative traces of the voltage-clamp recordings showing age-dependent sensitivity of DLG neurons to OXA (time of application pointed by the red bar). (B) Calculation of the percentage of the responsive cells for each age group, with a fit of quadratic curve (a + b × PD + c × PD^2^; a = 0.02 ± 0.26, *t*_9_ = 0.076, *p* = 0.94, b = 0.12 ± 0.038, *t*_9_ = 3.21, *p* = 0.011, c = −0.0049 ± 0.0012, *t*_9_ = −3.89, *p* = 0.0037, *n* = 12). The highest sensitivity to OXA was measured as 0.77 at PD12.42. (C) For the age groups PD4-19, we also analyzed the amplitude of the response to OXA (PD22 and PD25 were excluded due to a very low number of responses), shown here as current density. A linear mixed effects model was fitted with PD as a fixed effect (*F*_9,27_ = 3.71, *p* = 0.0039, *n* = 94), and rat as a random effect (SD = 1.16). *Post hoc* analysis revealed significant differences between PD4 and 16 (*t*_84_ = −3.76, *p* = 0.014) and between PD15 and 16 (*t*_84_ = −3.64, *p* = 0.021). ∗*p* < 0.05, ∗∗*p* < 0.01.

To build on our earlier MEA recordings and to assess whether ontogenetic changes in the properties of DLG neurons contribute to their responsiveness to orexin, we made voltage-clamp recordings from these neurons and challenged them with 200 nM OXA (160 neurons prepared from 49 rats over PD4-PD25) (Figure 5A). With neurons clamped at −65 mV, peak responsiveness to OXA (measured as the proportion of responsive neurons) increased from PD4 to a zenith between PD12-PD16 (max = 0.77 at PD12.42) and decreased to low levels at PD25 (Figure 5B). In general, the amplitude of responses to OXA increased between PD12 and PD15 (Figure 5C). Interestingly, the rare responses to OXA recorded at PD4 were characterized by high amplitude; an effect most likely associated with the higher input resistance of neurons at this age. Collectively, from these recordings, we suggest that OXA-evoked increase in firing rate of DLG neurons is attributable to an inward current whose developmental profile is also restricted to around the time of eye-opening.

### Orexin receptor 2 expression follows a developmental pattern

**Figure 6:**
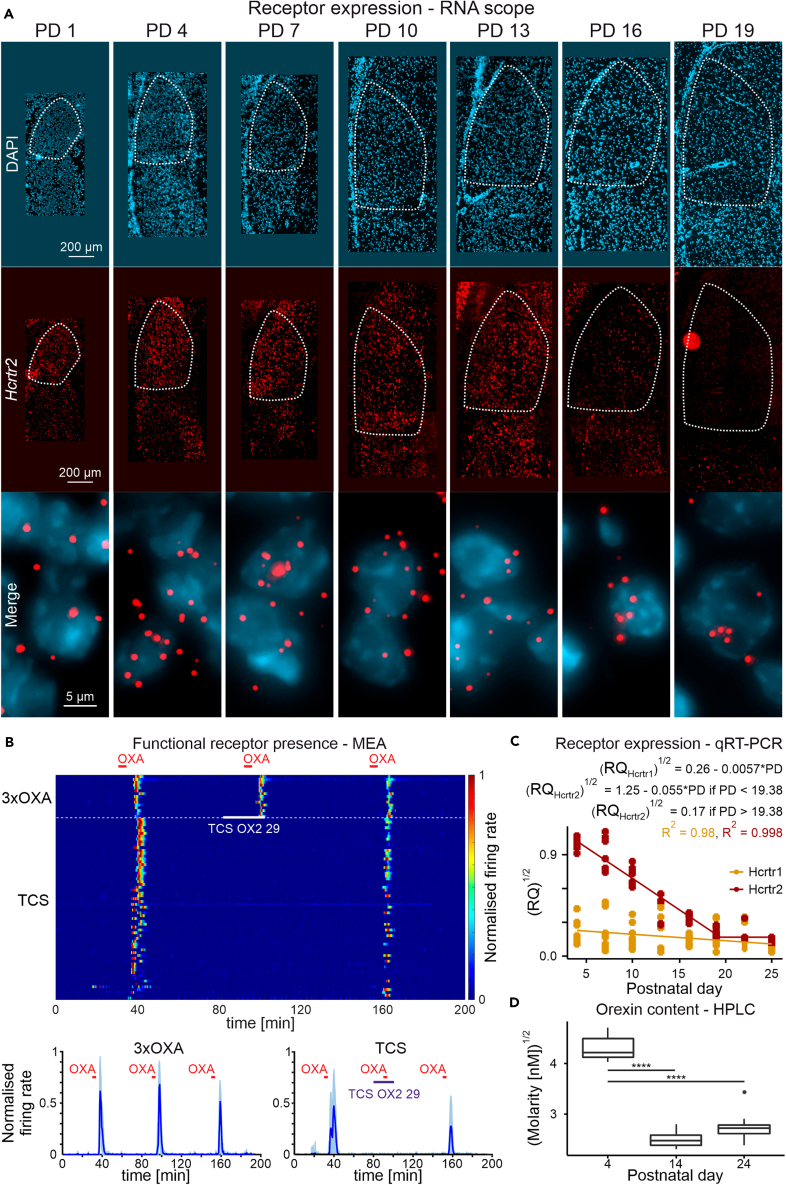
Orexin receptor 2 (*Hcrtr2*) expression follows a developmental pattern (A) Representative microphotographs showing developmental changes in *Hcrtr2* expression the DLG, and additionally, the notable differences in the size of this brain structure. (B) Temporal heatmap from the MEA experiments together with average activity plots, showing persisting responsiveness to three subsequent OXA applications (OXA1-OXA2: *t*_28_ = −1.64, *p* = 0.25; OXA1-OXA3: *t*_28_ = 1.33, *p* = 0.39; OXA2-OXA3: *t*_28_ = 5.22, *p* < 0.0001, *n* = 45) and its blockade during TCS OX2 29 (a selective OX_2_ receptor antagonist) application (OXA1-TCS: *t*_126_ = 13.08 *p* < 0.0001; OXA1-OXA3: *t*_126_ = 7.58, *p* < 0.0001; TCS-OXA3: *t*_126_ = −7.32, *p* < 0.0001, *n* = 192). (C) Age-dependent changes in the expression of *Hcrtr2* were analyzed by fitting a linear plateau mixed effects model in the form of a + b ∗x when x < jp, and a + b ∗ jp when x > jp (jp-junction point; a = 1.25 ± 0.039, *t*_36_ = 32.3, *p* < 0.0001, b = −0.055 ± 0.0031, *t*_34_ = −17.91, *p* < 0.0001, jp = 19.38 ± 0.76, *t*_34_ = 25.4, *p* < 0.0001, *n* = 73), with rat included as a random intercept (SD = 0.083). The expression of *Hcrtr1* decreased linearly (a = 0.26 ± 0.042, *t*_36_ = 6.2, *p* < 0.0001, b = −0.0057 ± 0.0027, *t*_35_ = −2.11, *p* = 0.042, SD_rat_ = 0.11, *n* = 73). However, *Hcrtr1* expression was generally very low. (D) Orexin content in the DLG decreased with age (*F*_2,9_ = 155.83, *p* < 0.0001, SD_rat_ = 0.13, *n* = 36), with significant differences between P4 and both other groups (P4-P14: *t*_9_ = 16.09, *p* < 0.0001; P4-P24: *t*_9_ = 14.33, *p* < 0.0001), but not between P14 and P24 (*t*_9_ = −1.76, *p* = 0.24). ∗∗∗∗*p* < 0.0001. See also Figure S4.

Our previous electrophysiological study raised the possibility that orexin receptor 2 (OX_2_ receptor) is functionally expressed in the DLG to mediate the actions of OXA and OXB on thalamocortical neurons within this structure.^15^ To obtain greater neuroanatomical precision of the development of OX_2_ receptor expression in this thalamic region, we next visualized its transcript (*Hcrtr2*) on the single cell level. We utilized RNAscope technology (fluorescent *in situ* hybridization) on rat brain tissue collected from 21 animals across PD1, 4, 7, 10, 13, 16 and 19 (*n* = 3 per group). At all ages examined, we detected *Hcrtr2* expression which was robust in young animals (PD1-13) but declined noticeably at PD16 (Figure 6A). Since *Hcrtr2* mRNA is translated to a functional protein, responses to OXA in the DLG should be blunted or abolished by an OX_2_ receptor antagonist. Consequently, we made additional MEA recordings from the DLG (4 thalamic slices prepared from 4 animals aged PD13-16) and evaluated whether a selective OX_2_ receptor antagonist TCS OX2 29 alters DLG responses to OXA. In slices in which OXA was applied three times, DLG neurons showed robust and replicable increases in firing rate, whereas in slices in which TCS OX2 29 (20 μM) was given prior to and during a second treatment with OXA, responses were abolished. Following 1 h for washout, responsiveness to OXA returned to baseline in these slices (Figure 6B). Based on these recordings, we argue that at this point in postnatal development, OX_2_ receptors are functionally expressed in the DLG.

To better quantify the developmental profile in OX_2_ receptor expression in the DLG, we next prepared thalamic slices from 37 rats at PD4, 7, 10, 13, 16, 19, 22, and 25 (n = 4–5 animals per group) and used RT-qPCR to measure *Hcrtr2* in the DLG. Linear-plateau curve fitting revealed that *Hcrtr2* expression was robust at PD4 and diminished linearly to very low levels at around PD19 (jp = 19.38 ± 0.76, *t*_34_ = 25.4, *p* < 0.0001, *n* = 73). By contrast, expression of *Hcrtr1* (which codes for the other orexin receptor; OX_1_) was and remained minimal in the DLG throughout postnatal development (Figure 6C). Subsequent visualization of *Hcrtr1* expression with RNAscope revealed that it was lacking in the *Hcrtr2-*rich DLG but was detectable in the IGL/VLG (Figure S4). To assess whether orexin is present in the postnatal DLG, we collected DLG tissue from 12 rat pups (*n* = 4 per group) at PD4, PD14, and PD24 and used HPLC analysis to quantify OXA. OXA was detected in all samples tested at the nanomolar concentration, with the highest values detected in the youngest animals (*F*_2,9_ = 155.38, *p* < 0.0001, *n* = 36; Figure 6D). Therefore, the presence of OXA and *Hcrtr2* expression co-occur early in postnatal development of the rat DLG with *Hcrtr2* expression diminishing around PD19.

### Blockade of OX_2_ receptors around the eye-opening day impairs thalamic visual responses in adults

**Figure 7:**
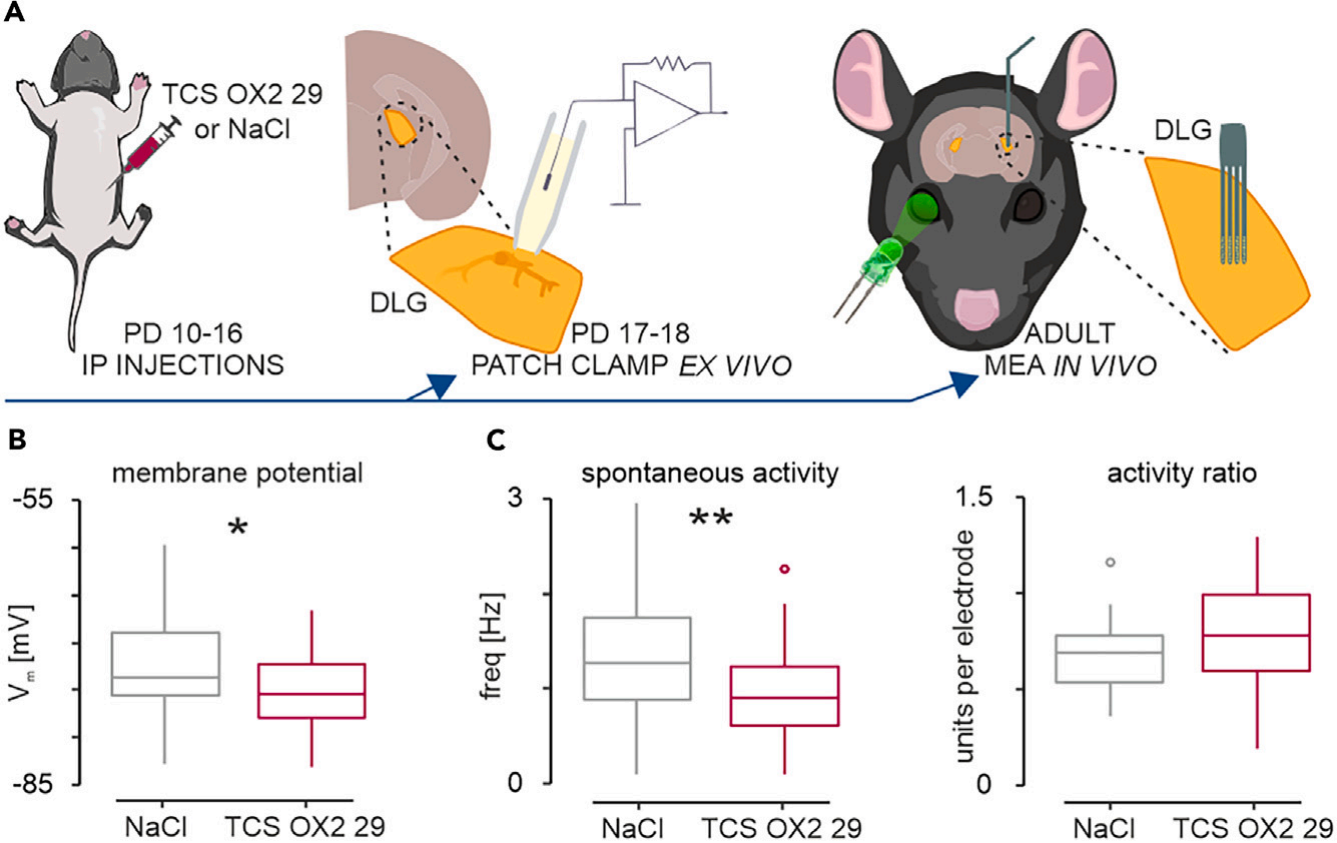
Blockade of OX_2_ receptors around the eye-opening day impairs spontaneous neuronal activity in the DLG (A) Schematic diagram of the experiment setup with segregation of saline (NaCl)- and TCS OX2 29 (OX_2_ receptor antagonist)-injected pups. Pups were assigned to one of two protocols: *ex vivo* patch-clamp at postnatal days (PD)17–18, or *in vivo* MEA in adulthood. (B) For patch-clamp experiments we fitted a linear mixed effect model, with treatment (NaCl vs. TCS OX2 29) as a fixed factor (*t*_5_ = −2.58, *p* = 0.049, *n* = 87) and both PD (17/18) and rat as random intercepts (SD_PD_ = 1.049, SD_rat_ = 0.00024). Due to the highly hyperpolarized membrane potential of DLG cells in slices, which does not permit spontaneous action potential firing, only the membrane potential was measured *ex vivo*. ∗*p* < 0.05. (C) In the *in vivo* experiment DLG cells fire spontaneous action potentials, therefore we analyzed differences in the spontaneous activity of the DLG neurons (*F*_1,14_ = 16.24, *p* = 0.0012, SD_rat_ = 0.12, *n* = 264) as well as activity ratio, defined as the number of recorded units/the number of electrodes within the borders of the DLG, for each animal (*F*_1,14_ = 0.14, *p* = 0.71, *n* = 16). ∗∗*p* < 0.01. See also Figure S5.

The visual system undergoes multiple postnatal developmental changes in distinct time frames which are defined as critical periods. Altering or impairing such changes within critical periods can result in irreversible loss of visual function in adulthood.^2^ Therefore, we next assessed if blockade of orexin-OX_2_ receptor signaling *in vivo* during the week around the day of eye-opening (coincident with when OXA most potently excites DLG neurons), affects light-evoked neuronal responses in the adult DLG. To do this, we gave daily i.p. injections of TCS OX2 29 or saline in 24 rat pups between PD10 and PD16. To assess the immediate effect of this blockade, in one cohort, (8 rats; 4 injected with OX_2_ receptor antagonist and 4 with saline) thalamic brain slices were prepared on PD17 or PD18 and patch-clamp recordings made to assess the fundamental properties of DLG neurons and their response to OXA. The remaining animals (*n* = 20) progressed to adulthood (between 250 g and 350 g; 10–12 weeks old) at which point they were subjected to *in vivo* recordings (Figure 7A).

Patch-clamp experiments *ex vivo* revealed that compared to saline-injected controls, the membrane potential of DLG neurons in TCS OX2 29 injected rats at PD17 or 18 was significantly hyperpolarized (*t*_5_ = −2.58, *p* = 0.049, *n* = 87; Figure 7B). Other neurophysiological parameters (e.g., membrane resistance, capacitance, and various measures of membrane excitability) were not overtly altered by a blockade of the orexinergic signaling, although rheobase was higher in TCS OX2 29 injected animals (Figure S5). This indicates impairing orexin signaling early in postnatal development acutely alters DLG neuronal excitability.

Since OXA affects the DLG in a precritical period for adult vision, when pruning and strengthening of retinogeniculate synapses occurs,^2^^,^^30^ we next evaluated if retinogeniculate transmission of information in adult rats *in vivo* was affected by blockade of orexin-OX_2_ receptor signaling. We used 32 channel MEA technology and recorded neuronal activity *in vivo* from deeply anesthetized rats (*n* = 16) who had either received TCS OX2 29 (*n* = 8) or control saline injection (*n* = 8) during postnatal development. Consistent with the hyperpolarized state of TCS OX2 29 treated PD17/18 DLG neurons reported above, the spontaneous activity recorded from the adult DLG in TCS OX2 29-injected rats was lower than in saline controls (*F*_1,14_ = 16.24, *p* = 0.0012, *n* = 264; Figure 7C). Further, the number of neurons monitored at recording electrodes in the DLG did not differ by treatment (*F*_1,14_ = 0.14, *p* = 0.71, *n* = 16; Figure 7C). From this observation we suggest that OX_2_ receptor blockade early in postnatal development results in long-term hyperpolarization of DLG neurons.

**Figure 8:**
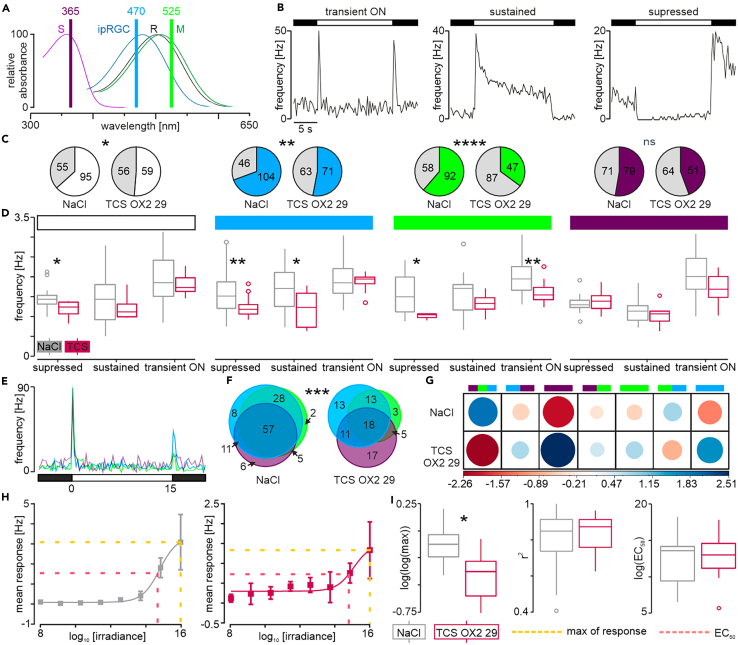
Blockade of OX_2_ receptors around the eye-opening day impairs thalamic visual responses in adults (A) Graph presenting the wavelengths of light used in the experiment together with absorbance spectra for different photoreceptors: S-cones, R-cones, M-cones, and intrinsically photosensitive retinal ganglion cells (ipRGCs). (B) Representative traces of the three light response types. (C) Frequencies of responses to each type of stimulation were analyzed with a chi-squared test. TCS OX2 29 treatment significantly reduced DLG neurons’ sensitivity to white (χ^2^_1_ = 3.87, *p* = 0.049), blue (χ^2^_1_ = 8.02, *p* = 0.0046), and green (χ^2^_1_ = 19.77, *p* < 0.0001), but not to UV light (χ^2^_1_ = 1.81, *p* = 0.18). (D) Amplitudes of the responses to each type of stimulation were analyzed with a linear mixed effects model, separately for each type of response. Treatment (saline - NaCl vs. TCS OX2 29) was included as a fixed factor and rat as a random intercept. The responsiveness to white light was shown to change for the suppressed cells (*F*_1,9_ = 7.79, *p* = 0.021, SD_rat_ = 0.12, *n* = 28), but not for the sustained (*F*_1,9_ = 1.76, *p* = 0.22, SD_rat_ = 2.5e-5, *n* = 37), or transient ON groups (*F*_1,8_ = 0.24, *p* = 0.64, SD_rat_ = 0.21, *n* = 31). For blue light, differences in the amplitude of the response were observed for the suppressed (*F*_1,12_ = 18.81, *p* = 0.001, SD_rat_ = 1.18, *n* = 75), as well as sustained types of responses (*F*_1,7_ = 5.89, *p* = 0.046, SD_rat_ = 1.48e-5, *n* = 27), but not for the transient ON (*F*_1,9_ = 0.02, *p* = 0.88, SD_rat_ = 3.91e-6, *n* = 24). TCS OX2 29 treatment affected responsiveness to the green light stimulation in the suppressed (*F*_1,8_ = 9.31, *p* = 0.016, SD_rat_ = 0.18, *n* = 42), and transient ON groups (*F*_1,11_ = 10.52, *p* = 0.0078, SD_rat_ = 0.12, *n* = 36), but not sustained (*F*_1,8_ = 1.4, *p* = 0.27, SD_rat_ = 0.22, *n* = 22). Last, responsiveness to the UV light remained unchanged in all groups (suppressed: *F*_1,9_ = 0.14, *p* = 0.72, SD_rat_ = 0.13, *n* = 28; sustained: *F*_1,7_ = 1.02, *p* = 0.35, SD_rat_ = 0.21, *n* = 26; transient ON: *F*_1,9_ = 1.88, *p* = 0.2, SD_rat_ = 0.27, *n* = 41). (E) Representative traces showing responses of an individual cell to different light wavelengths. (F) Proportional Venn diagrams presenting numbers of neurons sensitive to each light wavelength, including cells responsive to more than one type of stimulation. Differences in their frequencies between the treatment groups were analyzed with a chi-squared test (χ^2^_6_ = 26.4, *p* = 0.00019). (G) Residual plot from the chi-squared analysis, indicating major differences between the treatment groups. Most significant changes were observed in the frequencies of neurons sensitive to UV light-only and blue light-only (more in the TCS OX2 29 group), as well as the cells responsive to all three wavelengths (more in the control group). (H) Example of responses to increasing white light intensity in control and TCS OX2 29-treated rats. Neurons were classified as ‘light intensity-coding’ as their responses were fitted with a Hill’s curve. Yellow dotted line depicts maximal response, whereas red dotted line the EC_50_. (I) Coding parameters were analyzed with a linear mixed effects model, with treatment as a fixed factor and rat as a random intercept. A difference in the maximal response was observed (*F*_1,5_ = 7.57, *p* = 0.04, SD_rat_ = 2.68e-6, *n* = 16), but not in the R^2^ of the fit (*F*_1,6_ = 0.21, *p* = 0.66, SD_rat_ = 3.33e-6, *n* = 18) or EC_50_ (*F*_1,6_ = 0.00, *p* = 0.998, SD_rat_ = 0.00025, *n* = 19). ∗*p* < 0.05, ∗∗*p* < 0.01, ∗∗∗∗*p* < 0.0001. See also Figure S6.

To test if orexin-OX_2_ receptor blockade in postnatal development affected the processing of sensory information in the adult DLG, we next examined neuronal responses to retinal illumination through exposing the retina contralateral to the recorded DLG to a series of light pulses (duration: 15 s). First, polychromatic cold white light was used to broadly characterize light-evoked changes in firing rate. Subsequently, monochromatic blue, green, and ultra-violet (UV) light pulses were presented to the same eye, to activate melanopsin expressing intrinsically photosensitive retinal ganglion cells (ipRGCs), M cones, and S cones, respectively (Figure 8A). Overall, three distinct types of visual responses were recorded in the DLG: (1) a transient ON response characterized by peak of activity at the beginning and at the end of the stimulus, with no notable increase in firing throughout the light pulse; (2) a sustained response during which DLG neurons increased their neuronal activity throughout light stimulus; and (3) suppressed response elicited from neurons inhibited by the light pulse but producing an excitatory rebound of activity with the cessation of the stimulus (Figure 8B).

Using a polychromatic white light stimulus, the number of responsive DLG neurons was significantly reduced in animals treated with TCS OX2 29 in development (63.3% vs. 51.3% responsive, χ^2^_1_ = 3.87, *p* = 0.049, *n* = 265; Figure 8C). Within the light-responsive neurons from these animals, the amplitude was notably reduced for the suppressed response (*F*_1,9_ = 7.79, *p* = 0.021, *n* = 28; Figure 8D), but not for sustained (*F*_1,9_ = 1.76, *p* = 0.22, *n* = 37; Figure 8D) or transient ON responses (*F*_1,8_ = 0.24, *p* = 0.64, *n* = 31; Figure 8D). Next, in response to the ipRGC-activating blue light stimulus (associated with sustained responses), TCS OX2 29 treated rats showed reduced sensitivity with a significantly lower percent of responsive cells (69.3% vs. 53.0% responsive, χ^2^_1_ = 8.02, *p* = 0.0046, *n* = 284; Figure 8C), compared to control saline treated animals. Additionally, the amplitude was significantly blunted for suppressed (*F*_1,12_ = 18.81, *p* = 0.001, *n* = 75; Figure 8D) and sustained responses (*F*_1,7_ = 5.89, *p* = 0.046, *n* = 27; Figure 8D), while transient ON responses were not affected (*F*_1,9_ = 0.024, *p* = 0.88, *n* = 24; Figure 8B). Using a green light stimulus, known to evoke mainly transient ON responses in the DLG, revealed a reduction in the number of light-responsive cells in TCS OX2 29-treated animals (61.33% vs. 35.82% responsive, χ^2^_1_ = 19.77, *p* < 0.0001, *n* = 284; Figure 8C). Similarly, the amplitude of responses was decreased in light-suppressed neurons (*F*_1,8_ = 9.31, *p* = 0.016, *n* = 42; Figure 8D) as well as transient ON-activated ones (*F*_1,11_ = 10.52, *p* = 0.0078, *n* = 36; Figure 8D). No differences were seen in the amplitude of the sustained response to green light (*F*_1,8_ = 1.4, *p* = 0.27, *n* = 22; Figure 8D). Finally, we tested neuronal responses in the DLG to the retinal illumination with UV light, mostly activating the S cones. The i.p. injections with TCS OX2 29 during the precritical period in the development did not affect any of the response characteristic as both response frequency and amplitude did not differ between experimental and control groups. These findings provide evidence of long-term consequences of orexin-OX_2_ receptor blockade for DLG responsiveness and the development of retino-thalamic synapses processing green and blue but not UV light.

The ratio of DLG cells exhibiting a certain type of response did not differ between control animals and those injected with orexin receptor antagonist, when responses to white, blue, and UV light were considered. However, when tested with green light, the DLG of TCS OX2 29-injected rats produced significantly less sustained responses, in favor of transient ON ones (Figure S6). Moreover, the blockade of orexinergic signaling during the development changed the ability of DLG neurons to respond to multiple wavelengths (Figure 8E). In control conditions a vast majority of DLG units were responsive to two or all three light types tested, with a small number of cells specifically responding to blue, green, or UV light only. This was significantly changed in TCS OX2 29-injected rats where DLG neurons responsive to all wavelengths tested were as prevalent as those exclusively responsive to a particular light stimulus (χ^2^_6_ = 26.4, *p* = 0.00019; Figures 8F and 8G). Overall, this dataset suggests that both appropriate response type and the ability to respond to multiple wavelengths are altered by the blockade of OX_2_ receptor during postnatal development of the DLG.

Last, we evaluated the ability of DLG neurons to code light intensity, using white light stimulations over a range of intensities. Amplitudes of responses were calculated and fitted with Hill’s curve (Figure 8H). Neither the fit (*r*^*2*^*; F*_1,6_ = 0.21, *p* = 0.66, *n* = 18; Figure 8I) nor the EC_50_ (*F*_1,6_ = 0.00, *p* = 0.998, *n* = 19; Figure 8I) were affected by TCS OX2 29 injections during development. However, as expected, the maximal response was significantly blunted in DLG of rats subjected to OX_2_ receptor blockade (*F*_1,5_ = 7.57, *p* = 0.04, *n* = 16; Figure 8I). Since light intensity coding is performed at the level of the retina, we posit that the OX_2_ receptor blockade treatment protocol used here did not alter this function of the retina and that the altered responses to the different wavelengths of light are mediated at the level of DLG.

## Discussion

This study reveals an unexpectedly concise developmental window for orexin action on rodent thalamocortical DLG neurons, maximal around the day of eye-opening. The robust OXA-evoked depolarization is mediated by OX_2_ receptor activation, which transcription is already high in the first days of postpartum and linearly drops to a minimum before the end of third postnatal week. Additionally, our data demonstrate the concurrent neurophysiological maturation of DLG neurons, reaching adult parameters by PD18. Finally, our results provide compelling evidence that the activation of OX_2_ receptors in the precritical period around eye-opening is crucial for the appropriate responses of DLG neurons to light in the adulthood, particularly in the green and blue spectrum. These experiments are the first to directly show transient effects of the orexinergic system in the postnatally developing brain and consequently we propose a new role for this pleiotropic system. Interestingly, we find these effects to be specific to the developing visual system, with no similar transient developmental responsiveness to OXA in the auditory thalamus.

The orexinergic system of the lateral hypothalamus influences several physiological, behavioral, and pleiotropic processes including promotion of arousal such as active food seeking, motivation, reward, and wakefulness.^31^^,^^32^ This is in part due to the widespread projections of orexin neurons to the rest of the brain including the thalamus.^17^^,^^33^ An early study reported that the orexinergic system modulates activity of non-specific thalamocortical projection neurons, but has no effect on specific sensory relay thalamic neurons.^21^ However, our previous study found that orexins excite the majority of rat specific thalamocortical DLG neurons.^15^ Based on the new results provided by this current study showing a distinct window of the DLG responsiveness to orexins (ending in the middle of the previously studied age range), we believe that the discussed discrepancy between our previous patch-clamp work and those of Bayer and colleagues is attributable to differences in the age of the rats used (ours being younger). Indeed, other studies from our laboratory which were conducted on adult rats *ex vivo*^8^ and *in vivo*^16^ reported diminished sensitivity to orexins in the DLG.

The early postnatal responsiveness to OXA is paralleled and explained by an early functionality of the orexinergic system in the rodent development. Studies show that prepro-orexin is expressed in the fetal hypothalamus as early as ED12, followed by detectable levels of OXA at ED14.^23^ Similarly in human, OXA can be detected in the cerebro-spinal fluid in the third trimester of pregnancy.^34^ Anatomical and molecular development of orexinergic neurons (the number and size of cells, peptide expression) is completed by PD7 and in many ways the orexinergic system in that early postnatal development resembles its adult stage.^24^ Therefore, it is not surprising, however understudied, that orexins play a role in postnatal development of different neuronal circuits. Our study shows that OXA is detectable in functional nanomolar concentrations in the DLG at PD4, surprisingly at higher levels than in PD14 or PD24. This suggests that orexin-containing axons innervate the primary visual thalamus in early postnatal development, potentially governing its dynamic changes in first weeks postpartum.

The orexin receptors, OX_2_ and OX_1_, are differentially expressed in the adult rodent brain. The LGN expresses orexin receptors in its ventral parts (the IGL and VLG), while the DLG shows sparse or undetectable levels of receptor mRNA and protein.^18^^,^^35^^,^^36^ Here, with the use of molecular techniques and electrophysiology, we provide compelling evidence for functional OX_2_ receptor expression in the DLG in early postnatal development. Our *in situ* hybridization and qPCR experiments show that OX_2_ receptor transcription is highest in first postnatal days and linearly deteriorates to undetectable values at PD19. This is consistent with a recent microarray study showing *Hcrtr2* expression in the young DLG disappearing in adults.^37^ Intriguingly, we also show that significant responses to OXA in the DLG are delayed by a few days relative to OX_2_ receptor expression; they appear around PD7, peak at PD13, and last till PD16. This time lag may be explained by the programmed delay in the translation of *Hcrtr2* mRNA or a time-shifted transport of the receptor protein to neuronal membrane. Alternatively, the OX_2_ receptor may be translated and available in the membrane concurrently with *Hcrtr2* transcription, however its electrophysiological actions are not detectable yet due to an absence of intracellular signaling pathway proteins or targeted ionic channels. This latter possibility seems more likely as the DLG drastically changes its profile of gene expression over the first few weeks of postnatal development.^3^

The DLG is a part of the primary visual pathway whose postnatal development represents a classic stimulus-dependent sensory system maturation. At the level of thalamus, development of visual system can be divided into several distinct periods. In mice, PD8-10 marks the end of retinotopic map refinement and eye-specific segregation of retinogeniculate synapses.^38^^,^^39^ Initially, a large number of synapses are formed (>20) at each DLG cell, that are later eliminated to leave one to three, and strengthened approximately 50-fold by the PD20.^2^^,^^40^ However, the most prominent synapse elimination and strengthening takes place from PD7, spanning the eye-opening at PD12-13, toward PD16; exactly coinciding with the narrow period of DLG sensitivity to OXA. By this point, called a ‘precritical period’, spontaneous retinal activity, not visual experience, drives these changes.^41^ This is followed by a critical vision-sensitive period, during which light deprivation causes irreversible damage to adult vision.^42^

Developmental coincidence of OX_2_ receptor expression, orexin-derived postsynaptic activation of DLG neurons, and the period of synaptic pruning and strengthening may have significant functional consequences. The importance of postsynaptic depolarization to coincide with presynaptic stimulation is the basic requirement for the long-term potentiation and therefore is crucial for the retinogeniculate synapse maturation.^30^^,^^43^^,^^44^ Here, we directly show that in mice pups OXA acts on the DLG neurons activated by the stimulation of optic tract, with increased responsiveness to optic input during the OXA treatment. Moreover, the phosphorylation of transmembrane AMPAR regulatory proteins (such as stargazin) or transcriptional regulators (such as *Mecp2*)^45^^,^^46^ in the precritical period is crucial for plasticity at the retinogeniculate synapse. As OX_2_ receptor activation leads to recruitment of protein kinases, orexins are good candidates to play a role in the process.

In addition to synaptic maturation, it cannot be ruled out that orexins are shaping the postsynaptic development of DLG thalamocortical cells. Our results and previous reports^47^ clearly show that postsynaptic neurophysiological development of DLG neurons occurs postpartum. Indeed, our PCA indicated that the overall maturation of DLG membrane properties lasts until PD18. This includes the developmental changes in parameters whose underlying mechanisms have been previously reported to be affected by orexins in the DLG, including the effects on potassium and calcium conductance.^15^ This is further supported by the lowered membrane potential and neuronal activity of DLG neurons following the *in vivo* blockade of OX_2_ receptors between PD10 and PD16.

The developmental role of the DLG modulation by the orexinergic system during the precritical period is yet to be fully established. Interestingly, rat pups whose OX_2_ receptor was blocked from PD10 to PD16 (three days before and three days after eye opening) significantly differed in the light responsiveness of DLG neurons in adulthood. Intriguingly, this procedure did not affect all types of light responses equally; sensitivity to blue and green light was heavily impacted by the treatment, whereas responses to UV light remained untouched. In mice, S-opsin (sensitive to UV light) expression begins just before birth whereas M-opsin (sensitive to green spectrum) expression does not occur until around PD7.^48^^,^^49^ This shift in development supports the hypothesis that lack of orexinergic drive during M-cone development disturbs the adult responsiveness of DLG cells to green, but not UV light, as S-cones are already functional at PD10-PD16. On the other hand, ipRGCs responsible for blue-light responses in the DLG (altered by the blockade of OX_2_ receptor in the precritical period) are photosensitive at birth.^50^ However, the possible segregation in the development of cone-bipolar-RGC-DLG pathways for different light wavelengths needs further studies.

Blockade of OX_2_ receptor most potently altered the amplitude and number of light-evoked suppressions of neuronal activity in the DLG, evoked by both green and blue light stimulations. This response polarity may suggest the involvement of local inhibitory neurons in the DLG. Unlike other thalamocortical centers, the DLG contains a population of GABAergic interneurons which compose approximately 10% of all neurons in this structure.^51^^,^^52^^,^^53^ GABAergic interneurons in the DLG receive monosynaptic input from RGCs and provide feedforward inhibition onto excitatory thalamocortical cells to sharpen receptive fields and improve contrast of retinogeniculate transmission.^54^ Interestingly, these cells migrate to the DLG postnatally and undergo drastic changes in the first week postpartum. DLG interneurons extend their arborizations and mature neurophysiologically (by both pre- and postsynaptic mechanisms) by the PD21.^53^ Our previous study showed that DLG interneurons also express orexin receptors.^15^ Thus, it is possible that OX_2_ signaling between PD10 and PD16 contributes to their postnatal development and synapse forming. This includes communication between RGC axons and interneurons, but also forming the feedforward inhibition between interneurons and thalamocortical cells. Any interruption of this process would likely result in changes in cellular inhibition in adults, as seen in our study.

Here, we also show that a blockade of orexinergic drive in the DLG during the period of retinogeniculate synapse strengthening results in the disturbances in adult ‘multitasking’ in color detection of DLG neurons. In control condition, the vast majority of DLG neurons respond to retinal illumination with all three light wavelengths tested. However, after OX_2_ receptor blockade in precritical period, responses to different light colors are much more segregated; DLG neurons become specialized leaving a vast majority of cells less sensitive to green and blue light. This sensitivity realignment may have significant consequences for visual processing, possibly affecting form, color perception and visual acuity.^2^ To our best knowledge, no visual disturbances have been reported in the OX_2_ receptor knock-out mice; this may be due to lack of appropriate behavioral testing focused specifically on visual performance in these animals, or compensatory mechanisms in their development when OX_2_ receptor expression is lacking throughout their life.

In conclusion, our study is the first to show temporally confined action of the orexinergic system on the developing neurocircuitry, which disturbances have clear consequences in adulthood. The functional expression of OX_2_ receptor is constrained to a week surrounding the day of eye-opening during the precritical period for visual development, a time of retinogeniculate synapse pruning and strengthening. Our data provide comprehensive evidence that orexinergic modulation of the DLG during this developmental window is important for correct thalamic responsiveness to light in adult animals, and thus to normal visual processing. Our results also provide a plausible explanation to an apparent controversy on the DLG responsiveness to orexins, which may pave new avenues of research on the developmental roles of the orexinergic system in general.

### Limitations of the study

To assess the effects of OX_2_ receptor blockage on the developing DLG, we performed i.p. injections with the selective antagonist. This pharmacological manipulation is therefore not DLG specific. Thus, the possibility remains that other developing brain centers that express OX_2_ receptor and project to DLG (including retina^55^^,^^56^^,^^57^) mediate some of the reported effects.

Lastly, even though the study was performed on both males and females, sexual differences were not examined. While the DLG is generally not considered a sexually diverse structure, differences in the kinetics of its development cannot be excluded. Our experimental design, although balanced in the number of animals of either sex present in each group, did not support such analysis, although when including sex as a random variable we did not observe any differences in the output of a model (data not shown).

## STAR★Methods

### Key resources table

**Table:** 

REAGENT or RESOURCE	SOURCE	IDENTIFIER
**Antibodies**		

ExtrAvidin®−Cy3™	Sigma	Cat#E4142; RRID: AB_11218764
rabbit anti-neuropeptide Y	Sigma	Cat#N9528; RRID: AB_260814
anti-rabbit AlexaFluor 647	Jackson Immuno-Research	Cat#711-606-152; RRID: AB_2340625

**Chemicals, peptides, and recombinant proteins**		

TCS OX2 29	Tocris	Cat#3371, CAS#1610882-30-8
Orexin A	Bachem	CAS#205640-90-0
Triton™ X-100	Sigma	CAS#9036-19-5
Normal Donkey Serum	Abcam	Cat#ab7475
Fluoroshield™ with DAPI	Sigma	Cat#F6057
Atropinum sulfuricum WZF 1%	Polfa Warszawa S.A.	ATC code: S01FA01
Ketamine	Biowet Pulawy	https://biowet.pl/produkty/ketamina-10/
Sedazin (xylazine)	Biowet Pulawy	https://biowet.pl/produkty/sedazin/
CellTracker™ CM-DiI Dye	Invitrogen	Cat#C7000
Morbital	Biowet Pulawy	https://biowet.pl/produkty/morbital/
RNaseZAP™	Sigma	R2020
Aerrane Isoflurane	Baxter	EAN code: 5909990961627
Urethane	Sigma	Cat#94300

**Critical commercial assays**		

RNAscope Multiplex Fluorescent V2 Assay	Advanced Cell Diagnostics	https://acdbio.com/rnascope-multiplex-fluorescent-v2-assay
ReliaPrep RNA Tissue Miniprep System	Promega	Cat#Z6010
High-Capacity RNA-to-cDNA™ Kit	Applied Biosystems	Cat#4387406
QIAquick PCR Purification KIT	Qiagen	Cat#28104
PowerUp SYBR Green Master Mix	ThermoFisher Scientific	Cat#4368577

**Experimental models: Organisms/strains**		

Long Evans rats, wt	locally bred, Institute of Zoology and Biomedical Research, Jagiellonian University in Krakow, Poland	N/A
C57BL/6J mice, wt	locally bred, University of Exeter, UK	N/A

**Oligonucleotides**		

*Hcrtr1 (primer)*	Qiagen	GeneGlobe ID: QT00194005
*Hcrtr2 (primer)*	Qiagen	GeneGlobe ID: QT00177625
*Gapdh (primer)*	Qiagen	GeneGlobe ID: QT01082004
*Penk (probe)*	ACD Bio	Cat No. 417431
*Hcrtr1 (probe)*	ACD Bio	Cat No. 444761
*Hcrtr2 (probe)*	ACD Bio	Cat No. 484571

**Software and algorithms**		

MultiChannel Experimenter	Multichannel Systems	https://www.multichannelsystems.com/software/multi-channel-experimenter
Spike2 v8.13, v10	Cambridge Electronic Design Ltd	https://ced.co.uk/products/spkovin
Multi Channel DataManager	Multichannel Systems	https://www.multichannelsystems.com/software/multi-channel-datamanager
MatLab vR2018a, vR2020b	Mathworks	https://www.mathworks.com/products/matlab.html
KiloSort	Pachitariu et al., 2016^58^	https://github.com/MouseLand/Kilosort
NeuroExplorer v5, v6	Nex Technologies	https://www.neuroexplorer.com/
Signal 5.07	Cambridge Electronic Design Ltd	https://ced.co.uk/products/signal
CorelDRAW	Corel Corporation	https://www.coreldraw.com/en/
KilosortPLX	Pachitariu et al., 2016^58^	https://github.com/madeleinea/KilosortPLX
ZEN 2.3. blue edition	Zeiss	https://www.zeiss.com/microscopy/en/products/software/zeiss-zen.html
R v4.0.4	R Core Team	https://www.r-project.org/
RStudio v1.4.1106	RStudio Team	http://www.rstudio.com/

### Resource availability

#### Lead contact

Further information and requests for resources and reagents should be directed to the lead contact, Lukasz Chrobok (lukasz.chrobok@bristol.ac.uk).

#### Materials availability

This study did not generate new unique reagents.

#### Data and code availability

• All data reported in this paper will be shared by the lead contact upon request.
• This paper does not report original code.
• Any additional information required to reanalyse the data reported in this paper is available from the lead contact upon request.

### Experimental model and study participant details

#### Ethical approval

All procedures were carried out in accordance with the 2010/63/EU of the European Parliament and the Council of 22 September 2010 on the protection of animals used for scientific purposes and according to the guidelines of the Polish Animal Welfare Act of 23 May 2012 (82/2012) and the UK Animal (Scientific Procedures) Act 1986. The experimental procedures received approval from the Local (Krakow) Institutional Animal Care and Use Committee (No. 18/2018, 349/2022), and the research ethics committee of the University of Exeter. All efforts were made to minimize animal suffering and reduce the number of animals used, based on ARRIVE 2.0 guidelines.

#### Animals

Most experiments in this study were conducted on Long Evans rats housed and bred at the Animal Facility of the Institute of Zoology and Biomedical Research, Jagiellonian University in Krakow, Poland. They were selected for this study due to their full pigmentation of the eye and well-developed visual system.^59^^,^^60^ Some studies were conducted on C57BL/6J mice (also due to their pigmentation) bred in the Animal Facility of the University of Exeter. Animals were housed under standard conditions, in a 12:12 light-dark cycle at 23°C and 60% humidity with food and water available *ad libitum*. Adult animals after weaning were housed in groups of 5-6 rats per cage.

#### OX_2_ receptor blockade

In total, 24 rats were divided into two groups and injected intraperitoneally with either TCS OX2 29 (a potent OX_2_ receptor antagonist (Tocris, UK), 10 mg/kg body weight diluted in 0.9% NaCl) or 0.9% NaCl once a day at Zeitgeber time (ZT)1 from postnatal day (PD)10 to 16. After this treatment rats were housed in home cages and their welfare was monitored by a trained technician. A group of 8 rats (4 experimental, 4 control) were taken for patch-clamp recordings immediately after the injection period (PD17 and 18), whereas the remaining 16 were bred until reaching adulthood and used in the *in vivo* electrophysiological protocols.

### Method details

#### Electrophysiology *ex vivo*

##### Tissue preparation

###### Rats

92 Long Evans rat pups of both sexes were culled at ZT1 at PD4, 7, 10, 11, 12, 13, 14, 15, 16, 19, 22, and 25. First, they were deeply anaesthetised by isoflurane inhalation (1 ml in the incubation chamber; Baxter, USA) and decapitated. Then, the brain was carefully removed from the skull and immediately immersed in an ice-cold slice-preparation (rat) Artificial Cerebrospinal Fluid (prACSF) composed of (in mM): 25 NaHCO_3_, 3 KCl, 1.2 Na_2_HPO_4_, 2 CaCl_2_, 10 MgCl_2_, 10 glucose, 125 sucrose, with addition of pH indicator, 0.01mg/l Phenol Red; osmolality∼290 mOsmol/kg. prACSF was continuously gassed with carbogen (95% O_2_, 5% CO_2_). The brains were then trimmed into blocks and forebrains were mounted on an ice-cold plate of a vibroslicer (Leica VT1000S, Heidelberg, Germany) and cut into 250 μm-thick coronal thalamic slices. Up to five slices were then sectioned and split into two hemispheres and transferred to the pre-incubation chamber where they were submerged and maintained in carbogenated recording (rat) ACSF (rrACSF) composed of (in mM): 125 NaCl, 25 NaHCO_3_, 3KCl, 1.2 Na_2_HPO_4_, 2CaCl_2_, 2 MgCl_2_, 5 glucose, with 0.01 mg/l Phenol Red (initial temperature: 32°C, cooled to room temperature) for at least 1 h prior to the recording.

###### Mice

19 C57BL/6J mice pups of both sexes were culled at ZT1 between PD7 and PD19 in three-day intervals. Following cervical dislocation and decapitation, the brain was immersed in an ice-cold, carbogenated preparation (mouse) ACSF (pmACSF) composed of (in mM): 95 NaCl, 1.8 KCl, 1.2 KH_2_PO_4_, 0.5 CaCl_2_, 7 MgSO_4_, 26 NaHCO_3_, 15 glucose, 50 sucrose, and 0.005mg/l Phenol Red. Then, a block of tissue containing the thalamus was cut on a vibroslicer (Leica, VT1200S, Germany) into 250 μm-thick coronal slices. Slices were rested in pmACSF for a further 30 minutes and later transferred to warm (initial temperature: 32°C, cooled to room temperature) recording (mouse) ACSF (rmACSF) composed of (in mM): 127 NaCl, 1.8 KCl, 1.2 KH_2_PO_4_, 2.4 CaCl_2_, 1.3 MgSO_4_, 26 NaHCO_3_, 5 glucose, 10 sucrose, and 0.005mg/l Phenol Red, for 1h prior to the recording.

##### Multi-electrode array (MEA) recordings *ex vivo*

Thalamic slices were transferred to the recording wells of an MEA platform and processed according to previously described protocols.^61^ Rat slices were positioned on 6 × 10 recording array of the perforated MEAs (60pMEA100/30iR-Ti, Multichannel Systems, Germany) mounted in the MEA2100 System (Multichannel Systems) in a way to record the activity from the DLG and the subjacent IGL (high spontaneous activity, used as a control for slice quality). Mouse slices were recorded using the MEA2100-Mini-120 System (Multichannel Systems) and 12 × 12 perforated MEAs (120pMEA100/30iR-Ti, Multichannel Systems), which enabled the capture of neuronal activity from the whole LGN, surrounding thalamic areas, and the hippocampus. Throughout the whole experiment, slices were perfused with fresh carbogenated ACSF (rmACSF or rrACSF, depending on species) at a flow rate of 1.7-2 ml/min. Recording temperature was maintained at 32°C. After the initiation of gentle suction, slices were allowed to acclimatise for 30 min prior to start of the recording.

Data were acquired with MultiChannel Experimenter software (Multichannel Systems) at sampling frequency of 20 kHz. Baseline firing activity was recorded for 30 min prior to application of drugs. Then, 100 × stock solution of OXA (working concentration 200nM or 1 μM; Bachem, Switzerland) was diluted in 6ml of fresh ACSF and bath applied. In cases where OXA was applied 2-3 times, the interval between applications was 1h. For acute applications, the drug exposure time was approximately 3 min. Tonic applications of OXA (100nM) were performed for 12 minutes (20ml applied). TCS OX2 29 (20μM, from 100 × stock solution) was diluted in 50ml of fresh rrACSF and applied 10 min pre-, 3 min during, and 12 min post-OXA (200nM) application.

##### Optic tract stimulation *ex vivo*

17 mouse slices were subjected to optic tract electrical stimulation with a bipolar electrode during the recording of neuronal activity with the MEA. The electrode was placed on the optic tract at the level of the VLG. Negative current pulses (200 μs, 500 μA) were delivered from the stimulation unit (DS2A-Mk.II, Digitimer Ltd, UK) controlled by a Micro1401 interface (Cambridge Electronic Design Inc., CED, UK) using the Spike2 v10 software (CED). Stimulation trains containing 50 pulses were delivered at 0.5 Hz. Trains of stimulations were delivered 10 min prior to, as well as 10, 20, and 30 min following (post) the initiation of the 12 min tonic OXA (100 nM) application.

#### Patch-clamp

Following the incubation period, rat slices containing the DLG were placed in a recording chamber that was mounted on the stage of a Zeiss Axioscope microscope (Zeiss Ltd, Germany), and were constantly perfused with fresh, carbogenated rrACSF (32°C, 2 ml/min). Neurons were then randomly selected within the border of the DLG under a 40 × magnifying objective (Zeiss) fitted with infrared differential interference contrast. Borosilicate glass pipettes pulled at a horizontal puller (resistance∼7 MΩ; Sutter Instruments, USA) were filled with intrapipette solution containing (in mM): 125 potassium gluconate, 20 KCl, 10 HEPES, 2 MgCl_2_, 4 Na_2_ATP, 0.4 Na_3_GTP, 1 EGTA, and 0.05% biocytin (pH=7.4 adjusted with 5 M KOH; osmolality ∼300 mOsmol/kg). To obtain whole-cell configuration, suction was applied by an Ez-gSEAL100B Pressure Controller (Neo Biosystem, USA). The signal was amplified by a SC 05LX (NPI, Germany) amplifier, low-pass filtered at 2 kHz, and digitised at 20 kHz. All stages of experiments were recorded with Signal 5.07 and Spike2 v8.13 software (CED). A liquid junction potential of −15 mV was added to the measured values of membrane potential.

At the beginning of each recording, a series of electrophysiological tests were performed to assess basic membrane properties across the DLG development. Membrane potential was measured in current clamp mode (holding current = 0 pA). Threshold, rheobase, and action potential count were calculated from current ramp (duration: 1 s, amplitude: 1 nA). Capacitance, membrane resistance, and gain were calculated from a current step protocol (from -150 pA to +150 pA, increment: 10 pA, duration: 0.5 s). The amplitude of T-type calcium current (I_t_) was measured with a voltage step protocol (from: -95 mV to -38 mV, increment: 3 mV, duration: 200 ms). All current clamp tests were initiated from the holding current which set the membrane potential to -65 mV (manual adjustment). No additional pharmacological agents for a full current isolation were used. All tests were analysed in Signal 5.07 (CED) using custom-made scripts.

OXA (200 nM, Bachem) was applied in 3 ml of fresh rrACSF after recording a stable baseline in voltage clamp mode (holding potential = -65 mV). Negative rectangular pulses (duration: 1 s, amplitude: 25 mV) were applied every 60 s throughout the recording. Responsiveness to drug administration was measured with a custom-written script in MatLab (version R2018a, MathWorks). Changes in the whole-cell current were deemed significant if they differed by more than 3 x SDs of the averaged baseline values.

##### Post-recording staining

To ensure the accurate localisation of each recorded neuron within the DLG, post-recording staining was performed, as described before.^15^ In brief, slices were fixed in the 4% paraformaldehyde (PFA) solution in the 1 M phosphate buffered saline (PBS) overnight. Then, they were permeabilised in 0.6% TritX-100 (Sigma) and 10% normal donkey serum (NDS; Abcam) diluted in PBS for 3h at room temperature. Following, sections were incubated with Cy3-conjugated ExtrAvidin (1:250, Sigma) and primary antibodies against neuropeptide Y (NPY, raised in rabbit, 1:8000, Sigma) at 4°C overnight. Subsequently, slices were incubated with anti-rabbit AlexaFluor 647-conjugated antisera (Jackson Immuno-Research) for 6h at room temperature and mounted on glass slides in Fluoroshield with DAPI (Sigma). Finally, sections were inspected at 10 × magnification on an epifluorescence microscope (Axio Imager.M2, Zeiss). Biocytin-filled cells were classified as the DLG thalamo-cortical neurons based on their multipolar, heavily arborized dendritic tree, and localisation dorsal to NPY-positive cells of the IGL.^15^

#### Fluorescent *in situ* hybridisation

##### Tissue preparation

21 rats aged from PD1 to PD19 (n=3 for each developmental group, sampled every 3 days) were deeply anaesthetised with an overdose of isoflurane (Baxter) and decapitated. Brains were immediately removed from the skulls and flash frozen over dry ice and stored at -80°C. Brains were then cut into 16 μm thick coronal slices at -20°C with a cryostat (Leica CM1950), thaw-mounted on Superfrost-Plus slides (Thermofisher, USA), and stored again at -80°C. Finally, slides were thawed and fixed in the 4% PFA in 0.1M PBS for 15 minutes, rinsed twice in fresh PBS, and dehydrated using increasing ethanol concentrations (50%, 70%, 100%, and 100%). Dehydrated slides were air dried, and slices were outlined with a hydrophobic barrier pen.

##### RNAscope protocol and imaging of neonatal brain slices

Directly after the tissue preparation, slices were processed with the RNAscope multiplex *in situ* hybridisation protocol (Advanced Cell Diagnostics—ACD, USA). In the first step, slices were incubated with protease IV at room temperature (RT) for 1 minute only (the incubation time was dramatically shortened compared to adult tissue due to low myelination). Following two PBS rinses, slices were incubated for 2 h at 40°C with probes binding to *Hcrtr1*, *Hcrtr2*, and *Penk*. Next, slices were rinsed with wash buffer (ACD) and signal was amplified in a four-step protocol terminating with fluorophore tagging (*Penk* with Atto488, *Hcrtr2* with Atto550, and *Hcrtr1* with Atto647). Finally, slices were rinsed twice with fresh wash buffer, air dried, and coverslipped with Fluoroshield containing DAPI (Sigma, Germany). The area containing the LGN was scanned at 20 × magnification with the epifluorescence microscope (Axio Imager.M2, Zeiss, Germany) and images were taken with the ZEN software (ZEN 2.3. blue edition, Zeiss).

#### Quantitative reverse transcription PCR

##### Tissue preparation

40 rats were culled at 8 developmental time points (n=5 per age group, PD4-PD25 in three-day intervals) at ZT1 by an overdose of isoflurane and decapitation. Brains were quickly extracted from the skull in the ice-cold prACSF (for composition see *2.4.1.*), oxygenated with 95% oxygen and 5% CO_2_. Brains were then trimmed and cut with a vibroslicer at 250 μm-thick coronal thalamic slices containing the LGN. Bilateral DLGs were manually dissected under the dissection microscope using a scalpel, flash frozen on dry ice and stored in -80°C. During the protocol, all surfaces and instruments were treated with RNaseZAP (Sigma, Germany) to minimise ribonuclease activity.

##### RNA extraction and RT-qPCR

Tissue was processed for RNA extraction with ReliaPrep RNA Tissue Miniprep System (Promega, USA). Extracted RNA in RNase-free water was then stored at -80°C. Next, the normalised amount of RNA for each sample was processed for reverse transcription using the High-Capacity RNA-to- cDNA Kit (Applied Biosystems, USA). cDNA was purified using QIAquick PCR Purification KIT (Qiagen, USA). Subsequently, cDNA was stored at -20°C until the qPCR. The RT-qPCR reaction was performed using PowerUp SYBR Green Master Mix (ThermoFisher Scientific, Lithuania) and StepOnePlus Real-Time PCR System (Applied Biosystems). To measure the expression of genes of interest, QuantiTect primer assays (Qiagen, Germany) *Hcrtr1* (GeneGlobe ID: QT00194005) and *Hcrtr2* (GeneGlobe ID: QT00177625) were used. *Gapdh* (GeneGlobe ID: QT01082004) served as a housekeeping gene. Results were then analysed according to the Livak method (2^−ΔΔCT^) with *Gapdh* as reference gene and presented as relative target gene expression (RQ), where RQ = 1 indicates the mean expression of *Hcrtr2* at PD4.

#### LC-MS/MS

##### Tissue collection

A total of 12 rats were culled at PD4 (n=4), PD14 (n=4), and PD24 (n=4) at ZT1 by overdose of isoflurane and subsequent decapitation. Brains were taken out of the skull and processed according to a protocol described in *2.6.1.* section.

##### Sample preparation and analysis

Tissue sample was accurately weight and placed in 1.5 ml microcentrifuge tube. The sample was extracted with 100 μl methanol/acidified water with 0.1% TFA (90/10 v/v) by vortex-mixing for 10 min. The supernatant was collected after centrifugation at 15,000 × g for 10 min. 10 μl supernatant was injected into the LC-MS/MS system for analysis. LC-MS/MS was performed according to our previously established method.^62^ In brief, QTRAP 4000 triple quadrupole linear ion trap mass spectrometer equipped with an electrospray ionization (ESI) in multiple reaction monitoring (MRM) mode was applied for the detection of OXA by monitoring the transitions: MRM1 (quantifier) m/z 713.3 → 858.6 and MRM2 (qualifier) m/z 713.3 → 854.1. LC separation was performed with reserved-phase Kinetex C18 column and mobile phase consisted of 0.1% formic acid in water and acetonitrile in gradient mode. The retention time of OXA was 2.82 min.

#### Electrophysiology *in vivo*

##### Anaesthesia and surgery

Male Long Evans rats (10–12-week-old, 250-300 g) were initially anaesthetised with urethane (intraperitoneal administration, 1.6g/kg body weight, diluted in saline; Sigma, Germany). The depth of anaesthesia was verified by the complete absence of the hind paw pinching reflex and strong attenuation of the corneal reflex. Additional doses of anaesthetic (10% of the initial dose) were administered to the animal if necessary. The animals were then tracheotomized to reduce respiratory movements. During the experiments, the body temperature was maintained at 37°C with the use of an automatic heating pad (temperature controller TCP-02, WMT, Poland). To ensure stable positioning of the head during experiments, the animals were mounted on the ear and incisor bars in a stereotaxic frame (ASI Instruments Inc. Warren, MI, USA). A midline scalp incision was made and the skin and soft tissues covering the bones were pulled to expose the sutures and temporal ridges. Craniotomies were performed to allow the implantation of an electrode for ECoG recordings and the insertion of the multichannel probe into the DLG. The dura was removed from the craniotomy-outlined surface of the brain and all exposed brain surfaces were covered with a small amount of paraffin oil (Sigma, Germany) to prevent the tissue from drying. The pupil of the eye contralateral to the recorded DLG was dilated with atropine (Atropinum sulfuricum WZF 1%, Polfa, Poland) to allow light stimulation and prevent the natural occurrence of pupil size oscillations during sleep.^63^^,^^64^^,^^65^ Additionally, the eye surface was covered with mineral oil to retain corneal moisture. Supplemental doses of ketamine (20 mg/kg, Biowet Pulawy, Poland) and xylazine (Sedazin, 2 mg/kg, Biowet Pulawy, Poland) were injected intraperitoneally^66^ before the recording of the DLG activity to stabilise ECoG – all protocols were carried out in a brain state of cortical slow wave activity (SWA) characterised by a dominant 1 Hz frequency.^67^^,^^68^

##### Recording

A multichannel recording probe (A4X8–10 mm-50-200-177-A32, Neuronexus, USA) coated with a fluorescent dye (CM-DiI; Invitrogen, UK) was gently positioned in the DLG using the following coordinates: 4.5-4.9 mm posterior to the bregma, 3.6-4.2 mm lateral to the bregma, 4-4.7 mm ventral from the cortex surface, using a hydraulic micromanipulator (model: MO-10; Narishige International Ltd., Japan). One recording per animal was performed and the 32-channel probe signal was amplified (gain 50), filtered (high-pass filter 0.05 Hz), digitised at 40 kHz, and acquired data stored on a hard drive using the OmniPlex D Neural Data Acquisition System (Plexon, Inc., USA). Concurrently, brain state was monitored with an ECoG recording through a wire electrode mounted on a stainless-steel screw (600μm diameter) positioned above the primary visual cortex contralateral to the recording site. ECoG signal was amplified 1000× using a CyberAmp 380 amplifier (Axon Instruments, USA) equipped with a preamplifier connected to Micro1410interface (CED), band-pass filtered between 0.5–30 Hz and stored on a hard drive using the OmniPlex D Neural Data Acquisition System.

##### Light stimulation

Each experiment began with a 30-minute adaptation to darkness in a Faraday cage covered with light-impermeable material, after which 15 minutes of baseline neuronal activity was recorded. Light pulses were delivered through a custom-built LED-based light source (light-emitting diode; Faculty of Physics, Astronomy and Applied Computer Science, Jagiellonian University in Krakow, Poland) connected to a Master-8 stimulator (A.M.P.I., Israel). Each light-emitting diode (LED; 5 mm; Opto-Supply Ltd., China) was positioned 4mm from the surface of the eye. The light stimulation protocol consisted of illumination of the retina contralateral to the recorded DLG. It included polychromatic white (cold) and monochromatic blue, green, and ultraviolet stimulations (470 nm, 525 nm, and 365 nm, respectively) presented in the darkness. Additionally, white light was applied in the range of 8 intensities (10^8^ to 10^15^ photons/cm^-2^/s^-1^). During each light intensity, 6 pulses of light were applied (duration: 15 s, interstimulus intervals: 15 s, interval to the next light intensity: 5 min).

##### Histological verification of recording site

At the end of each experiment, the animals were sacrificed by an overdose of barbiturates (Morbital, Biowet Puławy, Poland). The brains were extracted and fixed in 4% PFA in 0.1M PBS for a minimum of 48 hours. The brain tissue was cut at 50 μm thick coronal slices using a vibroslicer (Leica VT1000S, Germany). Slices containing the DLG were then imaged using an epifluorescence microscope (Axio Imager M2, Zeiss, Germany) and location of recording sites identified with the rat brain stereotaxic atlas^69^ to verify the location of the DLG in the tissue and reconstruct the location of neurons using CorelDRAW software (Corel, Canada).

### Quantification and statistical analysis

#### Electrophysiology *ex vivo*

Raw, unfiltered data from MEA electrodes localised in the DLG (determined by images taken *in situ* during the recordings) were subjected to spike sorting, as described previously.^8^^,^^70^ In brief, data were first exported to HDF5 files using Multi Channel DataManager software (Multichannel Systems). Then, the files were remapped and converted to DAT format using a custom-made MatLab (version R2018a, MathWorks) script. Subsequently, data were spike sorted with the KiloSort program.^58^ Results of spike sorting were transferred, remapped, and filtered (Butterworth band pass filter fourth order from 0.3 to 7.5 kHz), and converted to CED-64 files using a custom made MatLab script, then manually curated in Spike2 (Spike2 v10; CED) with the use of principal component analysis (PCA) and autocorrelation functions.

Neuronal responses to OXA application were calculated in 30 s bins with NeuroExplorer 6 (Nex Technologies, USA). A unit was classified as responsive if its single unit activity increased by three standard deviations (SDs) from its baseline mean value. The amplitude of these responses (ΔHz) was measured as the difference between maximal firing frequency (in a 30 s bin) evoked by drug treatment and the recorded baseline. Neuronal responses to the optic tract stimulation were calculated in 0.2 s bins in NeuroExplorer 6 with the use of peristimulus time histograms (PSTHs). Units were deemed responsive if their firing rate following the stimulation was higher than 3 x SD of pre-stimulation baseline.

#### Electrophysiology *in vivo*

The recorded .pl2 files (Plexon file format) were converted to binary format and spikes were detected and sorted using Kilosort software (KilosortPLX)^58^ in MATLAB environment (MATLAB version R2020b, MathWorks). Using custom-made MATLAB scripts, the spike sorting results were transferred to .smrx files (Spike2 file format) containing a bandpass-filtered signal (300–7500 Hz bandpass filter, Butterworth, fourth order). The signal from each recording electrode that was previously found to be located in the DLG was then manually inspected. If needed, the signal was further refined with the aid of autocorrelogram inspection and PCA, as well as a visual comparison of the sorted spikes and the raw signal. The curated data were imported to NeuroExplorer 5 (Nex Technologies, USA) and PSTHs (bin size = 0.1 s) of neurons responses to a given light pulse were created: 5 s of baseline, 15 s of stimulation, and 5 s of post-stimulation period. Neuronal activity data were transferred to a custom MATLAB script to detect transient ON, sustained, and suppressed types of responses to light and response parameters were calculated.

Responses were classified as transient ON if their level of activity during 0.5s after light onset (named here as a ‘peak window’) exceeded the threshold of the 3 x SD of the baseline. Responses were considered sustained or suppressed when their mean level of activity between the end of the ‘peak window’ and the end of stimulation was at least 1 x SD higher or lower than the baseline, respectively. The following activity parameters returned by the script were further analysed accordingly with a previously established label: mean activity during baseline, during ’peak window’, and throughout the stimulation time except for ‘peak window’; relative changes between baseline activity and activity during ‘peak window’ and/or whole stimulation except ‘peak window’. Neurons were defined as ‘light intensity-coding’ when the goodness of fit (R^2^) of the Hill’s curve was ≥ 0.5.^71^^,^^72^

#### Statistical analysis

Statistical analysis was performed in R (Version 4.0.4) and RStudio (Version 1.4.1106). To account for multiple observations from the same animal we fitted mixed effects models with the animal as a random intercept. For multiple recordings from the same cell (in the case of multiple OXA applications) we included it as both a random intercept and a random slope. Linear mixed-effects models were fitted with *nlme* package,^73^ whereas for linear-plateau and other non-linear mixed models we additionally used *nlraa*^74^ and *minpack.lm*^75^ packages. PCA was performed with a help of *factoextra* package^76^ and visualised with *ggfortify*.^77^ Outliers were identified with the boxplot method for outlier detection from the *rstatix* package,^78^ as well as by inspecting QQ-plots (*ggpubr*^79^). Assumptions for a linear model in the case of groups were checked with Shapiro-Wilk normality test from the *rstatix* package and Levene test for homoscedasticity from *car* package,^80^ as well as with residual plots’ inspection, whereas for continuous variables only residual plots were used. Highly heterogeneous variance, observed for the control vs. TCS OX2 29 groups in the *in vivo* experiments was accounted for by adding groups’ weights as a variance structure in a model. *Post hoc* analyses were performed with *emmeans* package^81^ and Tukey’s correction for multiple comparisons. Model parameters are presented as estimate ± standard error. For the electrophysiological parameters the range of values is additionally reported as group mean and 95% CI [lower limit, upper limit]. Values of the parameters transformed for the analysis come from back-transformation.
