# Supplementary material for: A novel developmental critical period of orexinergic signaling in the primary visual thalamus
Source: iScience. 2024 Jun 24;27(7):110352. doi: 10.1016/j.isci.2024.110352 (PMC11269934; doi:10.1016/j.isci.2024.110352)
Supplement: Document S1. Figures S1–S6 [file mmc1.pdf]

**iScience, Volume *27***

## Supplemental information

# A novel developmental critical period of orexinergic signaling in the primary visual thalamus

**Anna M. Sanetra, Jagoda S. Jeczmien-Lazur, Kamil Pradel, Jasmin D. Klich, Katarzyna Palus-Chramiec, Marcelina E. Janik, Sylwia Bajkacz, Gabriela Izowit, Christian Nathan, Hugh D. Piggins, Alessio Delogu, Mino D.C. Belle, Marian H. Lewandowski, and Lukasz Chrobok**

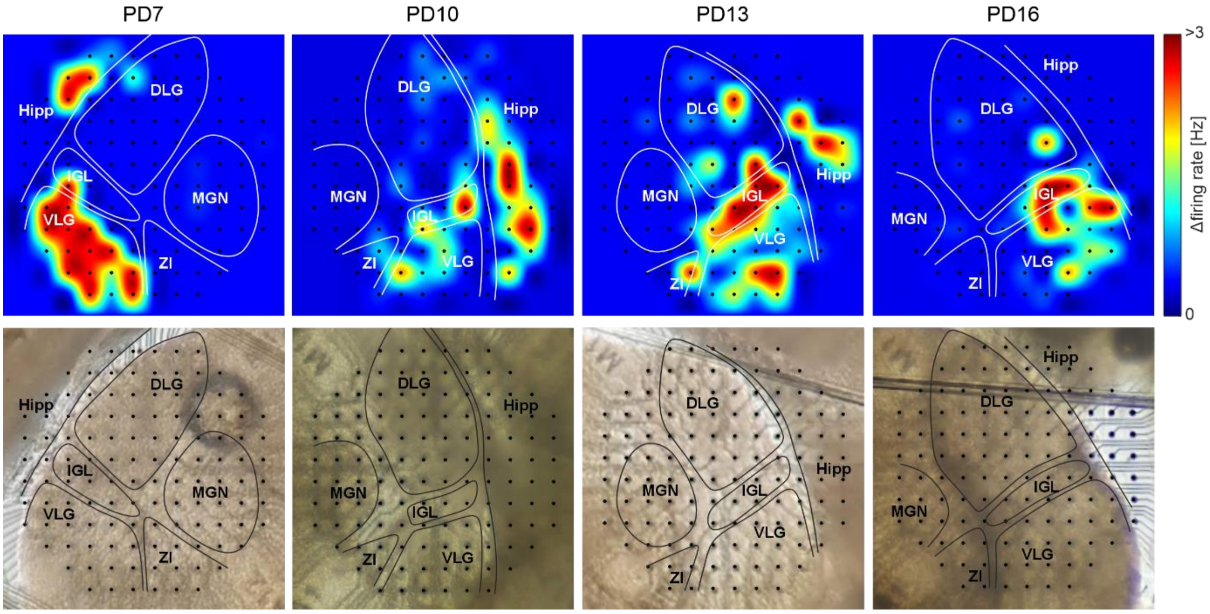


**Figure S1.** *OXA-evoked responses in mouse brain slices across early postnatal development.* Related to Figure 2.

Representative heatmaps (*above*) showing the amplitude of response to OXA (1 µM) throughout four developmental timepoints, with corresponding photographs depicting the positioning of a thalamic slice on a 120-channel MEA (*below*). Anatomical structures are delineated: DLG – dorsolateral geniculate nucleus, Hipp – hippocampus, IGL – intergeniculate leaflet, MGN – medial geniculate nucleus, VLG – ventrolateral geniculate nucleus, ZI – zona incerta. Please note, that no responses to OXA were recorded in the MGN throughout the developmental period, in which DLG sensitivity to OXA transiently occurs.

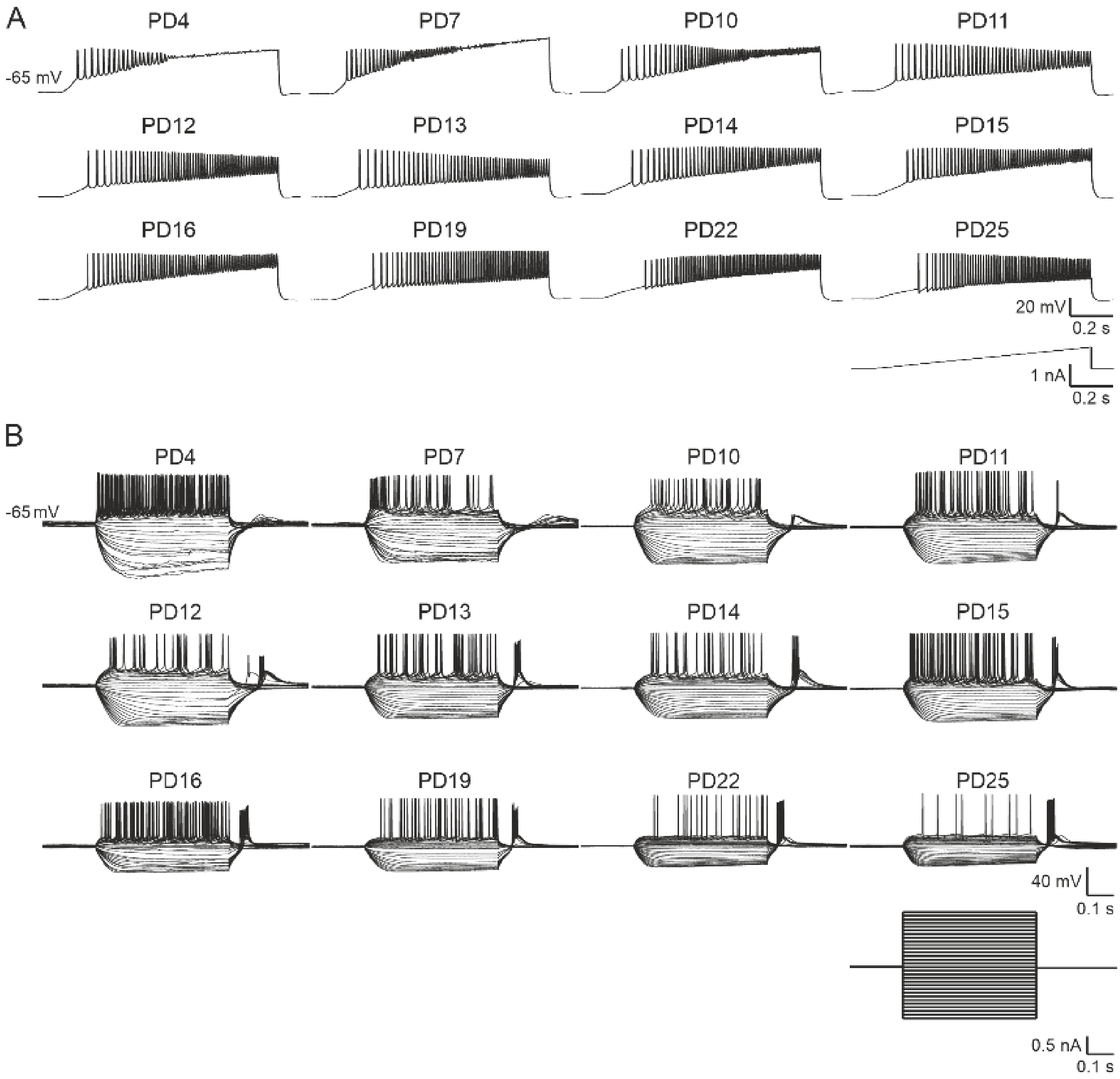


**Figure S2.** *Representative recordings of the response to a voltage ramp (A) and current steps (B) protocols from each age group*. Related to Figure 4.

A. The current ramp consisted of a single step of steadily increasing current up to a total amplitude of 1 nA within 1 s, after a manual adjustment of the membrane potential to -65 mV. From this protocol the threshold, rheobase, and action potential count were measured and compared between the age groups.

B. The current step protocol included 30 steps (from -150 pA to +150 pA, with 10 pA increment), each lasting 0.5 s and applied every 5 s, starting also from a membrane potential manually adjusted to -65 mV. This protocol enabled the calculation of the capacitance, membrane resistance, and gain.

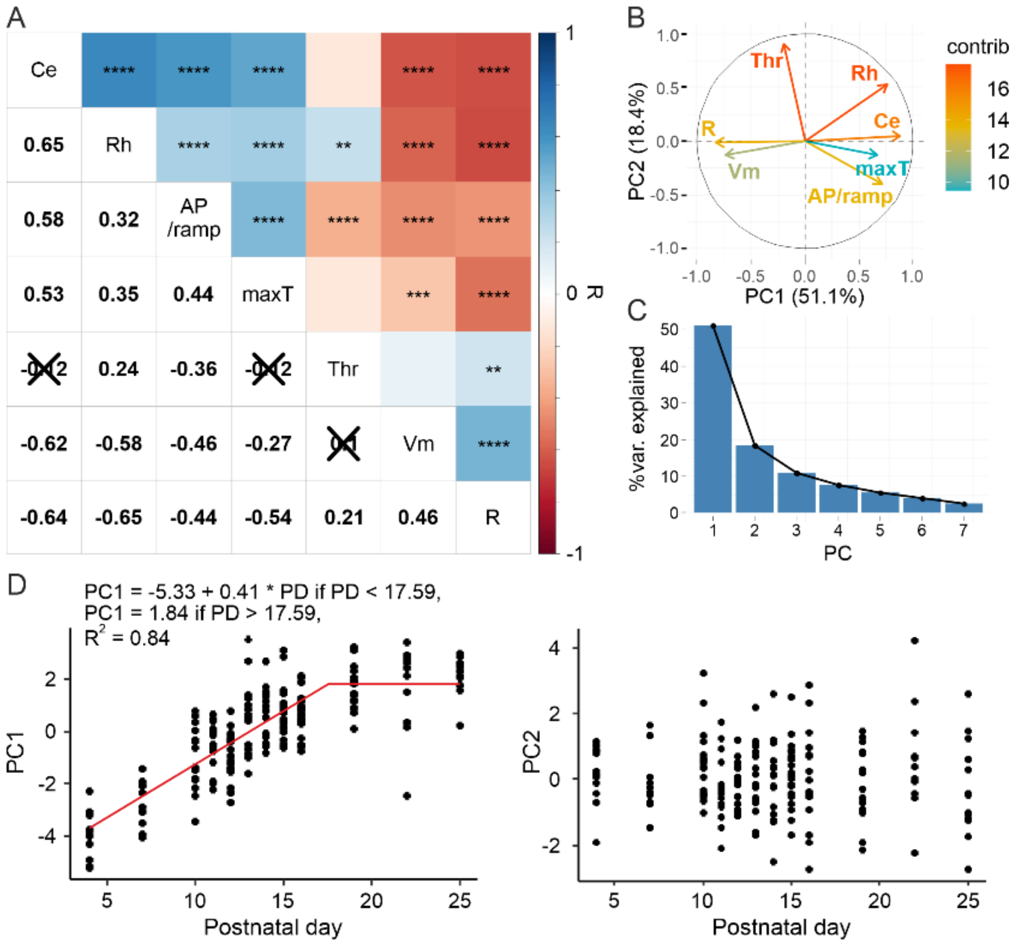


**Figure S3**. *The results of the principal components analysis (PCA) on the recorded electrophysiological parameters (excluding gain).* Related to Figure 4.

A. Correlation plot of the variables used for the PCA, showing highly significant correlations between majority of the parameters. Upper part shows significance level of the correlation (**p<0.01, ****p<0.0001) with a colour-coded correlation coefficient, whereas the lower part presents exact values of the coefficient. These found non-significant are crossed out.

B. Graph of variables indicating their contributions to the first two principal components.

Abbreviations: Vm – membrane potential, Thr – threshold, Rh – rheobase, Ce – capacitance, R – resistance, maxT – maximum value of the T-type calcium current

C. Scree plot of the eigenvalues of the principal components.

D. Analysis of the first two PCs was performed with a linear mixed effects model (rat as a random intercept). PC1 followed a linear-plateau fit in a form of a + b * x when x < jp, and a + b * jp when x > jp (jp - junction point; a=-5.33±0.46, $t_{132}$=-11.62, $p$<0.0001, b=0.41±0.038, $t_{47}$=10.84, $p$<0.0001, jp=17.59±0.86, $t_{47}$=20.36, $p$<0.0001, $SD_{rat}$=0.67, n=182), whereas PC2 did not change with age (a=0.077±0.25, $t_{132}$=0.3, $p$=0.76, b=-0.0053±0.017, $t_{48}$=-0.31, $p$=0.75, $SD_{rat}$=0.23, n=182).

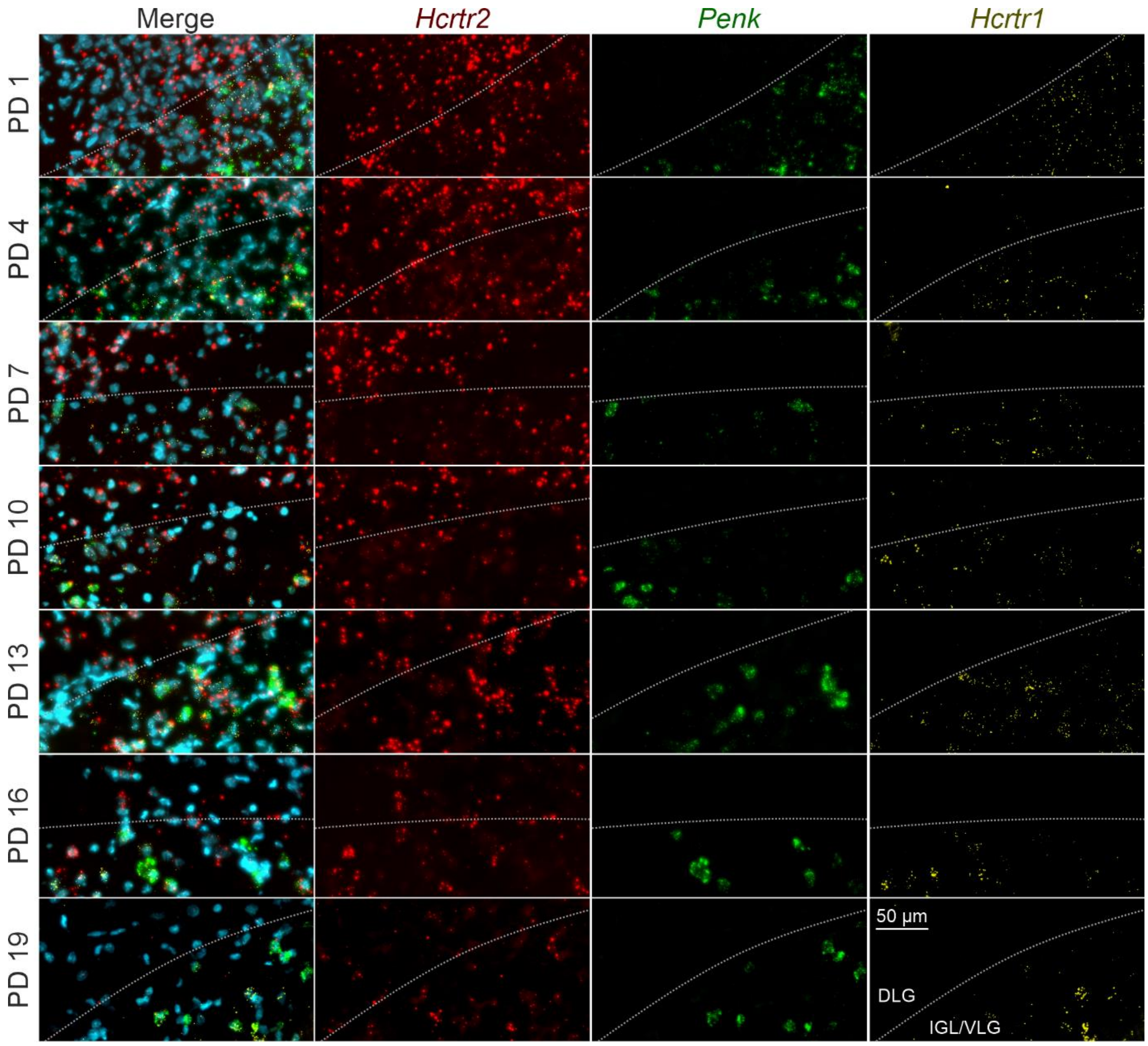


**Figure S4.** *Results of the RNAscope study showing spatial distribution of Hcrtr1 and Hcrtr2 in the lateral geniculate nucleus.* Related to Figure 6.

Representative microphotographs showing developmental changes in *Hcrtr2* expression. Hcrtr1 was shown to be confined to the area occupied by IGL/VLG. Border detection was aided with proenkephalin-expressing cells (*Penk*).

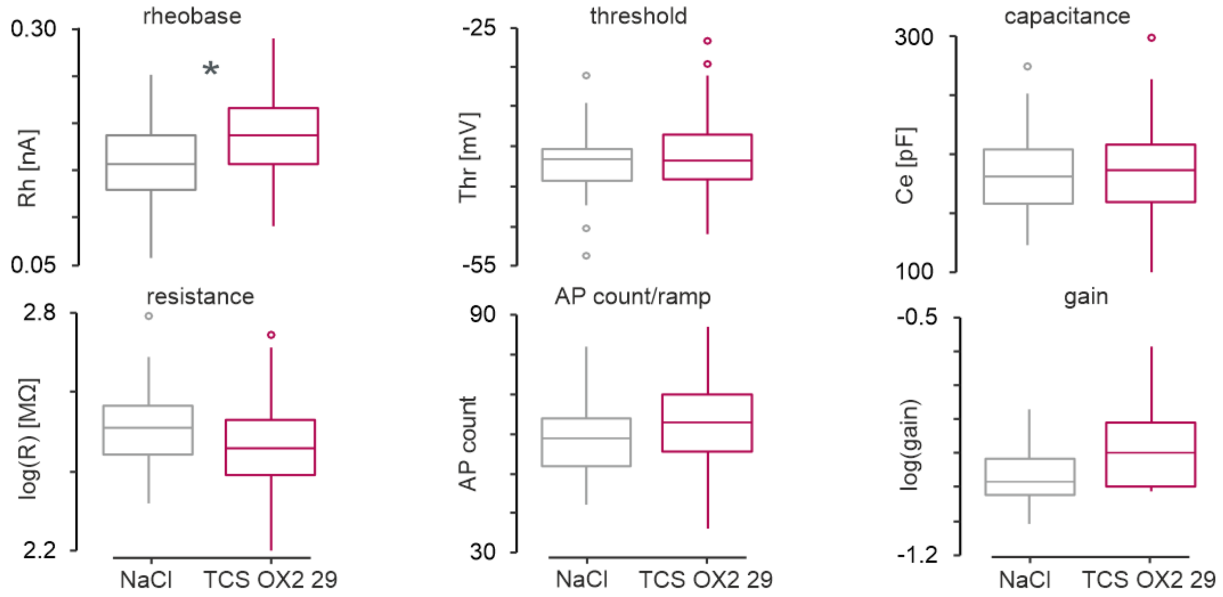


**Figure S5**. *Blockade of $OX_2$ receptors around the eye-opening day does not influence the majority of electrophysiological properties of DLG neurons.* Related to Figure 7.

For each recorded parameter we fitted a linear mixed effect model, with treatment (control - NaCl vs. TCS OX2 29) as a fixed factor and both PD (17/18) and rat as random intercepts. A significant impact of TCS OX2 29 treatment was observed for rheobase ($t_5$=3.32, $p$=0.021, $SD_{PD}$=0.019, $SD_{rat}$=1.09e-6, n=84), but not for threshold ($t_5$=1.13, $p$=0.31, $SD_{PD}$=1.2, $SD_{rat}$=1.28, n=87), capacitance ($t_5$=0.56, $p$=0.6, $SD_{PD}$=0.0026, $SD_{rat}$=0.017, n=84), resistance ($t_5$=-1.36, $p$=0.23, $SD_{PD}$=2.29e-6, $SD_{rat}$=0.027, n=83), action potential (AP) count ($t_5$=0.087, $p$=0.93, $SD_{PD}$=4.2, $SD_{rat}$=5.27, n=80), or gain ($t_5$=1.55, $p$=0.18, $SD_{PD}$=2.14e-6, $SD_{rat}$=0.063, n=37). *p<0.05.

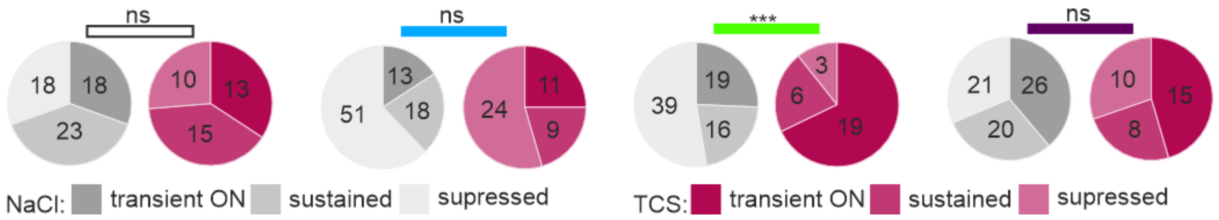


**Figure S6**. *Blockade of $OX_2$ receptors around the eye-opening day influences the types of responses to green light.* Related to Figure 8.

For each wavelength used for light stimulation, we analysed the frequencies of the different types of responses with a chi-squared test. No differences were observed for either white ($\chi^2_2$=0.24, $p$=0.89), blue ($\chi^2_2$=1.57, $p$=0.46) or UV light ($\chi^2_2$=0.49, $p$=0.78). However, a highly significant influence of TCS OX2 29 treatment was seen for the types of responses to green light ($\chi^2_2$=18.4, $p$=0.0001), with a decrease in the suppressed group in favour of the transient on group. ***p<0.001.
